# FBXW7α regulates amyloid pathology by mediating ubiquitination and degradation of BACE1 in Alzheimer’s disease

**DOI:** 10.1038/s41420-026-03159-y

**Published:** 2026-05-20

**Authors:** Yu Yang, Luping Jia, Jiachen Xu, Jiwen Wu, Haoyang Huang, Hewen Yang, Zihan Qi, Yixuan Wang, Hao Yu, Shuai Wang

**Affiliations:** 1 Shandong Key Laboratory of Psychiatric and Behavioral Medicine, School of Mental Health, Jining Medical University, Jining, China; 2 Shandong Collaborative Innovation Center for Diagnosis, Treatment and Behavioral Interventions of Mental Disorders, Jining Medical University, Jining, China; 3 Cheeloo College of Medicine, Shandong University, Jinan, China

**Keywords:** Alzheimer's disease, Alzheimer's disease

## Abstract

The dysregulation of proteostasis is a hallmark of Alzheimer’s disease (AD), characterized by the accumulation of misfolded and aggregated proteins. Dysfunction of the ubiquitin-proteasome pathway is a major contributing factor to proteostasis imbalance. The E3 ubiquitin ligase, F-box and WD repeat domain-containing 7 (FBXW7), a key hub factor in AD, is significantly downregulated in AD patients. FBXW7 mediates the proteasomal degradation of tau and regulates the development of tau pathology. However, the effect of FBXW7 on β-amyloid pathology and the underlying mechanisms remain unclear. This study demonstrated that FBXW7α, the dominant FBXW7 isoform, was localized in both the cytoplasm and nucleus of neurons. Aging led to a decline in FBXW7α protein levels in the brain tissues of both wild-type and 5×FAD mice. Notably, the level of FBXW7 in the brain tissue of 5×FAD mice is significantly lower than that in wild-type mice after 6 months of age. FBXW7α interacted with BACE1 via the conserved phosphodegron motif and targeted BACE1 for degradation. FBXW7 knockdown diminished the ubiquitination of BACE1, impaired its proteasome-mediated degradation, and increased the accumulation of BACE1 in Golgi fractions. Additionally, restoration of FBXW7α in the hippocampus improved cognitive function and ameliorated amyloid pathology in 5×FAD mice. Our findings suggest that FBXW7α acts as a key regulator of amyloid pathology, and highlight FBXW7α as a promising potential therapeutic target for AD intervention.

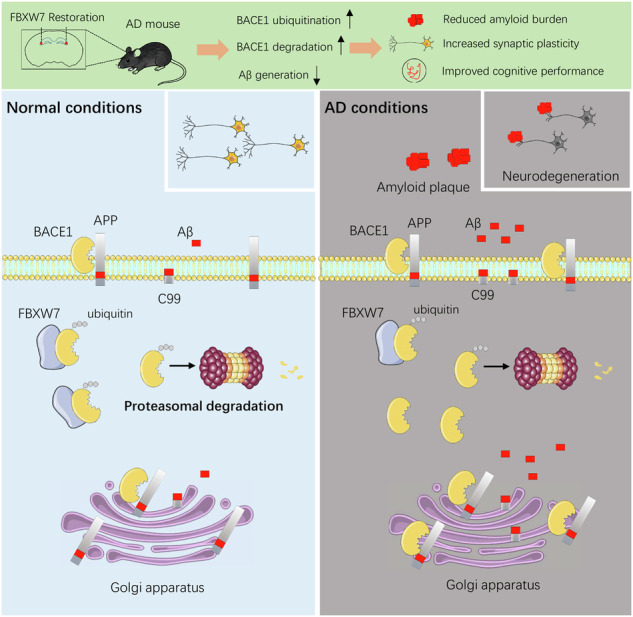

## Introduction

Alzheimer’s disease (AD) is a progressive neurodegenerative disorder characterized by the accumulation of amyloid-β (Aβ) plaques and neurofibrillary tangles composed of hyperphosphorylated tau protein in the brain. These pathological hallmarks contribute to synaptic dysfunction, neuronal loss, and cognitive decline, with pronounced deficits in learning and memory [1–3]. As the leading cause of dementia, AD currently affects more than 50 million people globally. Given the trend of population aging, this prevalence is projected to triple by 2050 [4]. Despite extensive research, the precise mechanisms underlying AD pathogenesis remain incompletely elucidated, underscoring the need for continued investigation into the molecular pathways that contribute to disease progression.

Protein homeostasis, commonly referred to as proteostasis, is critical for maintaining cellular functionality and is governed by an interconnected network of pathways that oversee protein synthesis, folding, and degradation [5, 6]. The dysregulation of proteostasis is a hallmark of AD, characterized by the accumulation of misfolded and aggregated proteins [7–9]. The ubiquitin-proteasome system (UPS) plays a critical role in maintaining proteostasis by selectively degrading damaged or unnecessary proteins [10]. Impaired UPS function has been linked to AD pathogenesis, as diminished proteasomal activity exacerbates the accumulation of toxic protein aggregates, further compromising neuronal degeneration [11, 12]. Therefore, elucidating the regulatory mechanisms of the UPS, particularly the role of E3 ubiquitin ligases, may inform the development of novel therapeutic interventions for AD.

The F-box and WD repeat domain-containing 7 (*FBXW7*) gene encodes a substrate-recognition component of the SKP1-CUL1-F-box (SCF) E3 ubiquitin ligase complex. FBXW7 exists in three major isoforms (α, β, and γ), which differ in their subcellular localization and tissue distribution, suggesting their distinct regulatory roles [13, 14]. FBXW7 targets several oncoproteins and cell cycle regulators for degradation, implicating it in tumor suppression [15, 16]. However, emerging evidence suggests that FBXW7 also regulates neurodevelopmental and neurodegenerative processes [17, 18]. Dysregulation of FBXW7 has been associated with neurological disorders, including AD [19–21], where its role in modulating proteostasis and mitigating protein aggregation warrants further exploration. Multi-omics analysis revealed FBXW7 as a central hub in AD pathophysiology, with multifaceted regulatory effects on the core pathological cascades of this disease [21, 22]. FBXW7 has been reported to be downregulated in AD patients [22] and mouse models [21]. Integrated transcriptomic analysis identified FBXW7 as a key signature gene associated with neuroinflammatory and synaptic dysfunction. FBXW7 directly binds to tau protein, mediating its polyubiquitination and proteasomal degradation in a phosphorylation-dependent manner. Enhancing hippocampal FBXW7 expression in vivo markedly reduces tau burden and rescues cognitive deficits in PS19 tauopathy mice [23]. However, the involvement of FBXW7 in the amyloid pathology associated with AD has not been fully elucidated.

In the present study, we demonstrate that the α isoform of FBXW7 (FBXW7α) is downregulated in neurons of 5×FAD. This reduction in FBXW7α expression inhibits the degradation of BACE1, leading to its accumulation and consequently promoting Aβ generation. Importantly, restoring FBXW7α level in 5×FAD mice ameliorates both Aβ pathology and cognitive deficits. Together, our findings identify FBXW7 as a critical regulator of Aβ pathogenesis in AD and propose a potential therapeutic strategy to mitigate the neuropathology of AD.

## Results

### FBXW7α localized to both the nuclear and cytoplasmic compartments in neurons

Previous studies have indicated that the *FBXW7* gene exhibited a tissue-specific expression profile, with *FBXW7α* and *FBXW7β* transcripts being particularly abundant in the brain [24, 25]. Nevertheless, FBXW7 expression within the neuronal lineage remains elusive. To detect the α- and β- subtypes of FBXW7 in neuronal cells, we chose six commercial antibodies targeting different immunogens within FBXW7 proteins (Fig. S1A). Several expression plasmids encoding various FBXW7 isoforms were constructed and overexpressed in N2a and HEK293 cell lines. Subsequent immunoblot detection revealed that five antibodies specifically recognized only the FBXW7α isoform, even though the immunogen sequences for antibody 1,3,5 and 6 were derived from conserved regions shared between the α and β isoforms (Fig. S1B-H). Following evaluation of the immunoblot outcomes, antibody 6 (purchased from Proteintech) was selected for subsequent experiments as it produced a clean signal which showed no significant affinity difference for murine and human FBXW7α proteins (Fig. S1G, H). To validate the expression of exogenous FBXW7 proteins, plasmids for expression of flag epitope tagged FBXW7 α and β isoforms were constructed and transfected into N2a cells. Consistent with prior findings, the FBXW7 antibody specifically detected only the α isoform. In contrast, anti-Flag immunoblotting identified two distinct bands, ~130 kDa and greater than 250 kDa in size, both of which are substantially larger than the predicted molecular weight of the β isoform (60 kDa) (Fig. S1I). The electrophoretic analysis using a 15% gel yielded consistent results, with no small fragment bands detected, indicating that the N-terminal flag remained intact and was not cleaved (Fig. S1J).

**Fig. 1:**
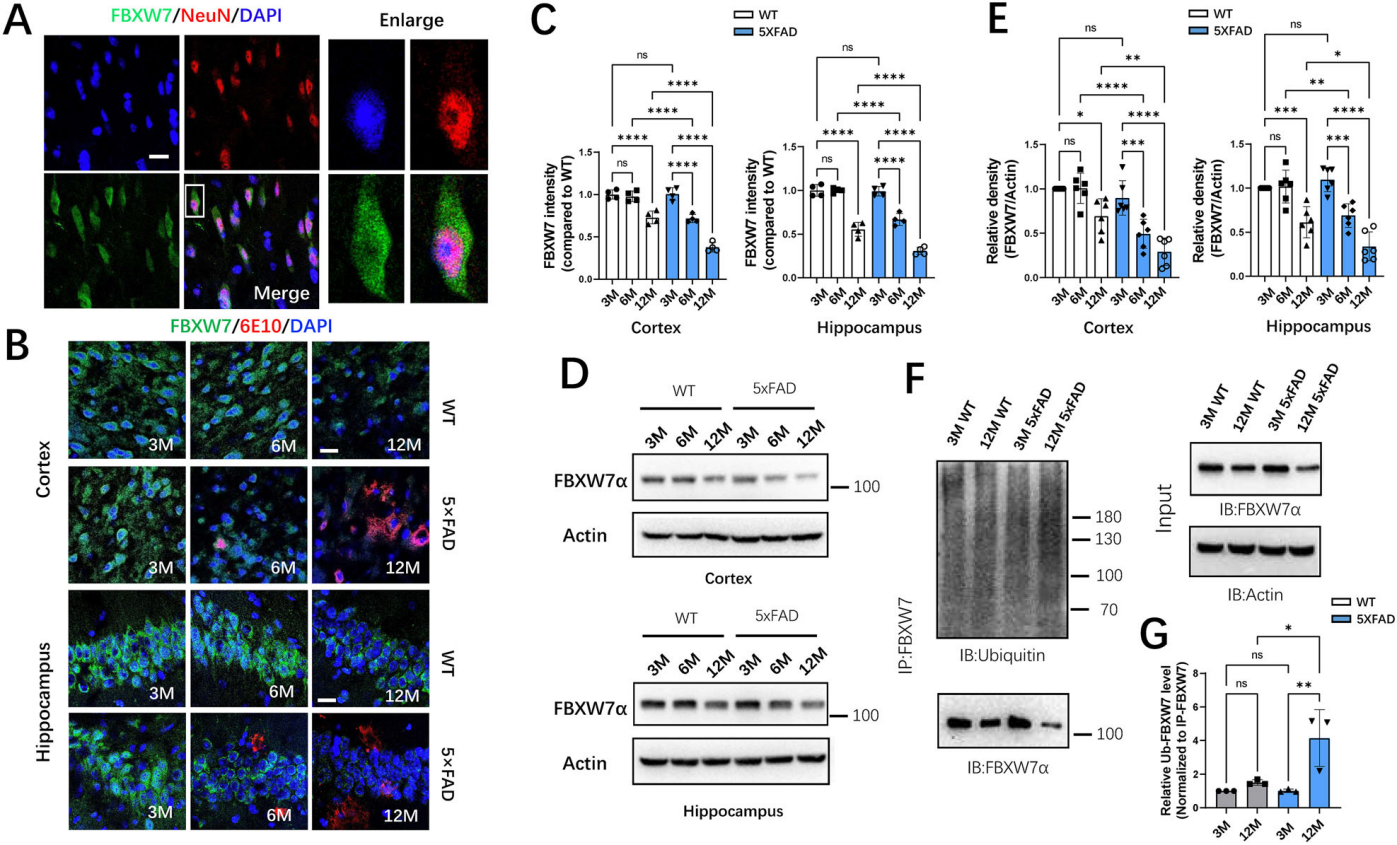
Aging induces FBXW7α reduction in the brain. **A** Immunofluorescence analysis of FBXW7α expression in cortical neurons of wild-type mice. Representative immunofluorescence images **B** and quantitative analysis **C** of FBXW7 expression in cortical and hippocampal regions of WT and 5×FAD mice at 3, 6, and 12 months of age (*n* = 4 per group). Representative immunoblotting images **D** and quantitative analysis **E** of FBXW7α expression in cortical and hippocampal tissues of WT and 5×FAD mice at 3, 6, and 12 months of age (*n* = 6 per group). **F**, **G** Immunoprecipitation detection of ubiquitinated FBXW7α protein in cortical tissues of 3- and 12-month-old WT and 5×FAD mice (*n* = 3 per group). Scale bar: 20 μm. Data show mean ± S.D, and each experiment was conducted with a minimum of three replicates. *P value < 0.05, **P value < 0.01, ***P value < 0.001, ****P value < 0.0001, ns, not significant by one-way ANOVA with Bonferroni correction.

To further elucidate the subcellular localization of the α and β isoforms of FBXW7, we expressed Flag-tagged FBXW7 in N2a cells and performed immunofluorescence staining using antibodies against FBXW7 and the Flag epitope. Consistent with the immunostaining results, the FBXW7 antibody can only specifically detected the α isoform, which exhibited a broad distribution in both the cytoplasm and nucleus (Fig. S2A). Moreover, we confirmed the localization of endogenous FBXW7α in brain slices (Fig. 1A, B) and cultured primary neurons (Fig. S2B), where it was also present in both compartments. In contrast, immunofluorescence analysis with the Flag antibody revealed that FBXW7β was predominantly localized in the cytoplasm (Fig.S2A). These findings demonstrate that FBXW7α is highly expressed in neurons and localizes to both the cytoplasm and nucleus, contrary to previous reports indicating its exclusive nuclear distribution in other cell types [26, 27].

### Aging triggers a decline in FBXW7α expression in both WT and AD mouse brains

FBXW7 has been reported to be downregulated in the brain tissues of AD patients [22, 28] and APP/PS1 transgenic mice [21]. However, the precise alterations of its expression remain unclear. In this study, we examined wild-type (WT) and 5×FAD mice at different ages and assessed FBXW7 expression levels in hippocampal and cortical tissues. Using an FBXW7 antibody previously validated for its specificity against the α isoform (Figs. S1 and S2), we performed immunofluorescence in brain slices of mice at different months of age, including WT and 5×FAD. The results demonstrated that FBXW7α is predominantly expressed in neurons (Fig. 1A), which is consistent with single-cell RNA sequencing data from the Human Protein Atlas database (https://www.proteinatlas.org/). Moreover, we observed an age-dependent downregulation of FBXW7α in both the cortex and hippocampus of WT and 5×FAD mice (Fig. 1B and C). Notably, compared to WT mice (6–12 months), the downregulation of FBXW7α in 5×FAD mice occurred earlier (0–6 months). Western blot analysis further corroborated these findings (Fig. 1D and E).

To investigate the mechanism underlying FBXW7α downregulation, we measured *FBXW7* mRNA levels. RT-PCR results showed no significant differences in the levels of *FBXW7α* or *FBXW7β* mRNA between WT and 5×FAD mice across age groups (Fig.S3), suggesting that the downregulation is regulated at the protein level. As an E3 ubiquitin ligase, FBXW7 is subject to degradation via the ubiquitin-proteasome pathway, and ubiquitination promotes its proteasomal turnover [29, 30]. Immunoprecipitation used by FBXW7 antibody followed by subsequent immunostaining against ubiquitin revealed that aging increased endogenous FBXW7α ubiquitination, an effect that was more pronounced in AD model mice (Fig. 1F and G). Specifically, 12-month-old 5×FAD mice exhibited significantly higher FBXW7α ubiquitination levels compared to age-matched WT controls, indicating that the AD condition enhances FBXW7α ubiquitination and subsequent protein degradation (Fig. 1F and G). In summary, these results demonstrate that aging promotes the downregulation of FBXW7α protein levels, and this effect is accelerated in an AD model context.

### FBXW7α regulates APP processing

The dysregulation of FBXW7α indicates its potential role in the pathogenesis of AD. To further investigate the functions of FBXW7α, we overexpressed FBXW7α in N2a cells and conducted quantitative 4D label free proteomic profiling. Comparative analysis revealed that 234 proteins were significantly up-regulated and 151 proteins were down-regulated in the FBXW7α-overexpressing group relative to the control (Fig.S4A and B). KEGG pathway enrichment analysis of these differentially expressed proteins indicated a strong association between FBXW7 and cancers, corroborating extensive previous studies [15, 31, 32]. Beyond its well-established role in cancer, our data also suggest that FBXW7α may be implicated in the pathogenesis of neurodegenerative disorders, particularly AD (Fig.S4C). Furthermore, protein domain enrichment analysis revealed a potential link between FBXW7α and the generation of the C-terminal fragment of the β-amyloid precursor protein (APP), implying a role for FBXW7 in the regulation of APP proteolytic processing (Fig.S4D).

**Fig. 2:**
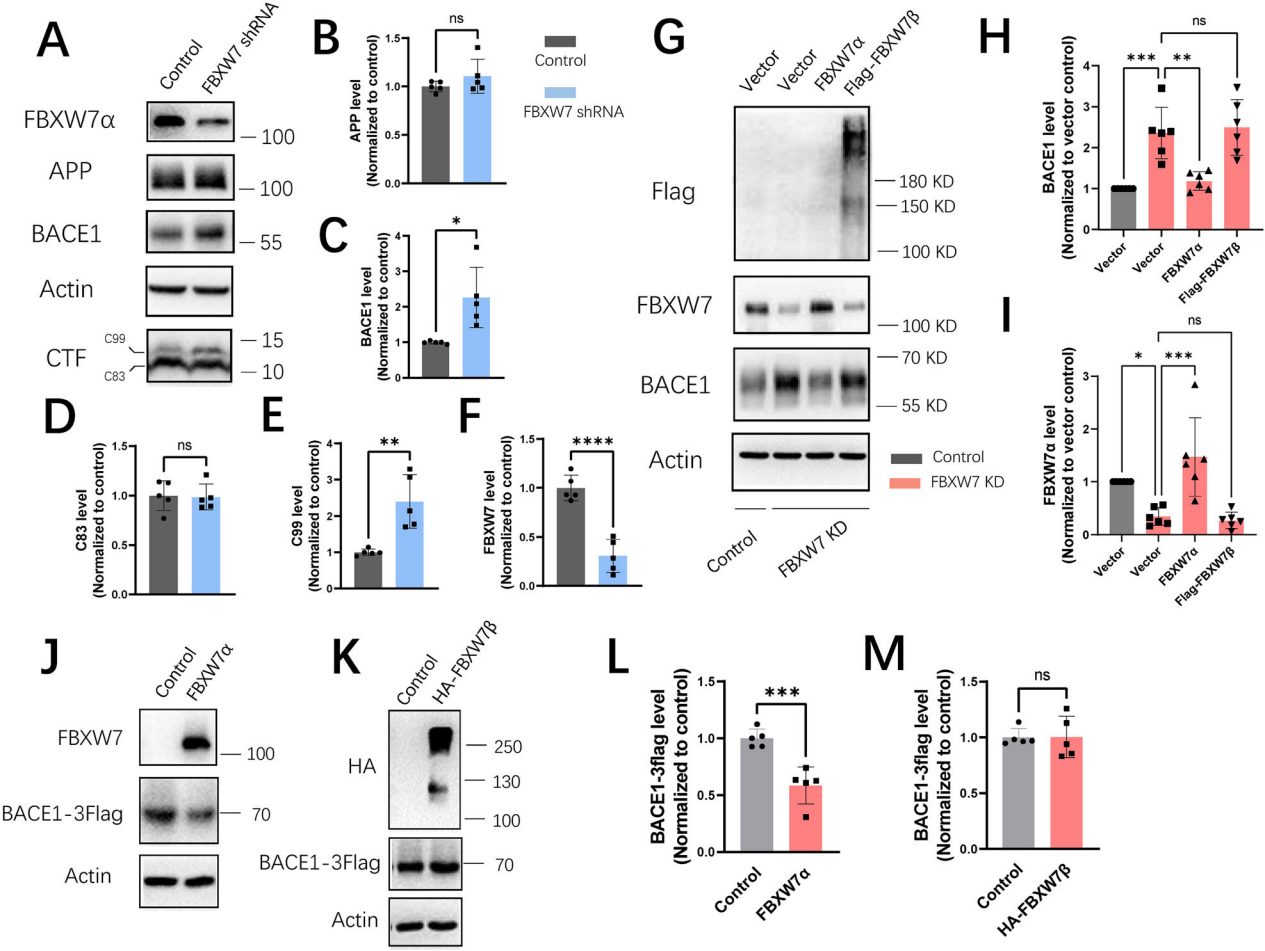
FBXW7α regulates BACE1 level and APP processing. Representative immunoblotting images (**A**) and quantitative analysis of APP (**B**), BACE1 (**C**), C83 (**D**), C99 (**E**), and FBXW7 (**F**) in N2a cells overexpressing Swedish mutant APP (*n* = 5 per group); FBXW7 knockdown was conducted using *FBXW7* shRNA delivered through transient transfection. Representative immunoblotting images (**G**) and quantitative analysis of BACE1 (**H**) and FBXW7α (**I**) in scramble control(Control) and FBXW7-knockdown stable (FBXW7 KD) N2a cells expressing FBXW7α or Flag-FBXW7β by transient transfection, respectively (*n* = 6 per group). Empty vector was used as control. Representative immunoblotting images (**J**, **K**) and quantitative analysis of BACE1 (**L**, **M**) in N2a cells overexpressing BACE1-3flag (*n* = 5 per group). FBXW7 overexpression was conducted using FBXW7α and HA-FBXW7β delivered through transient transfection. Data show mean ± S.D. *P* values were determined by Student’s t test (**B**–**F**, **L**, **M**) or one-way ANOVA with Bonferroni correction (**H**, **I**). *P < 0.05; **P < 0.01; ***P < 0.001; ****P < 0.0001; ns, not significant.

To clarify the regulatory role of FBXW7 in APP cleavage, we conducted FBXW7 knockdown by using FBXW7 shRNA in the N2a cell line overexpressing Swedish mutant APP. Western blot results demonstrated that the APP protein level remained unchanged, while BACE1 was significantly upregulated (Fig. 2A–C). Consistent with this finding, C99, the BACE1 cleavage product of APP, was also notably upregulated, suggesting that FBXW7 may influence the APP cleavage process by regulating the level of BACE1 (Fig. 2A, D-F). Furthermore, we also constructed FBXW7 knockdown stable N2a cell line. It was observed that the BACE1 protein level was upregulated (Fig.S4E and G), whereas its mRNA level showed no alteration (Fig. S4J). Neither the APP protein level nor its mRNA level exhibited any change (Fig.S4F and I). To investigate the different effects of FBXW7α and FBWX7β on BACE1 level, we performed rescue experiments by re-expressing either FBXW7α or FBXW7β in N2a cells with stable FBXW7 knockdown (Fig. 2G–I). The results showed that only re-expression of FBXW7α, but not FBXW7β, reduced BACE1 protein levels. Furthermore, we overexpressed FBXW7α or FBXW7β individually in cells stably expressing BACE1-3×Flag to determine their specific effects on BACE1 protein level. Consistent with the findings in Fig. 2(G–I), only FBXW7α overexpression significantly decreased BACE1 levels, whereas FBXW7β had no effect (Fig. 2J–M). Together, our results indicate that FBXW7α could affect the β-cleavage of APP by regulating the level of BACE1.

### FBXW7α mediates BACE1 degradation through the ubiquitin-proteasome pathway

**Fig. 3:**
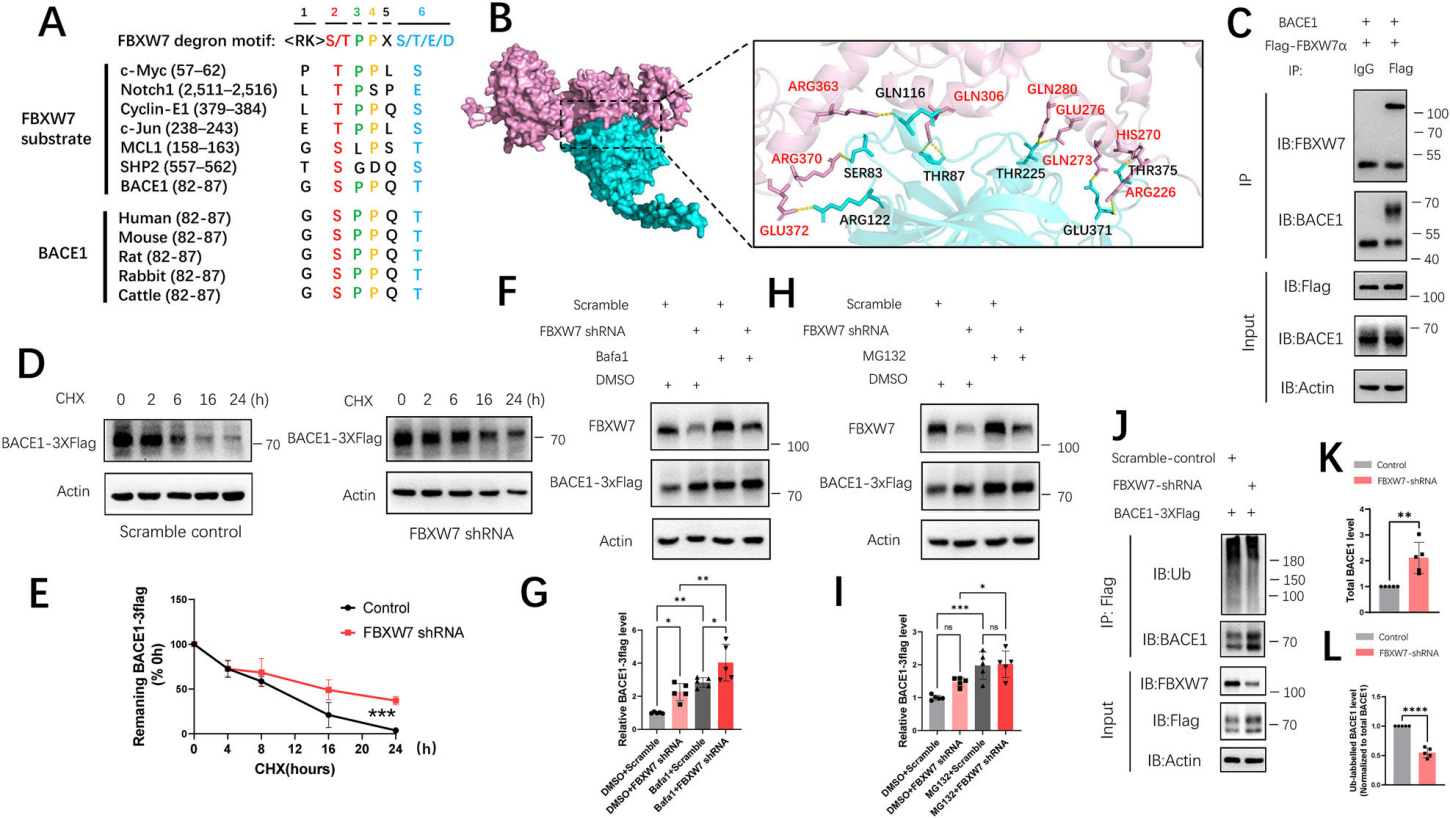
FBXW7α interacts with BACE1 and potentiate its proteasomal degradation. **A** Scheme showing the conserved sequences of FBXW7 degron within BACE1 and other reported FBXW7 substrates. **B** Interacting model of FBXW7α with BACE1 simulated by protein docking software. FBXW7α in Pink and BACE1 in cyan; Yellow dashed lines indicate hydrogen bonds. Interacted amino acids were shown in red or black color for FBXW7α and BACE1, respectively. **C** Co-immunoprecipitation determines the interaction of Flag- FBXW7α with BACE1. Representative immunoblotting images **D** and quantitative analysis **E** of BACE1-3×Flag level at different time course by CHX treatment in control and FBXW7 knockdown stable N2a cell lines (*n* = 4 per group). *P* values were determined by Two-way ANOVA with Bonferroni correction. *P < 0.05. Representative immunoblotting images (**F**, **H**) and quantitative analysis (**G**, **I**) of BACE1-3×Flag level in control and FBXW7 knockdown stable N2a cells treated by Bafa1 (**F**, **G**) for 6 h or MG132 (**H**, **I**) for 8 h, respectively (n = 5 per group). *P* values were determined by one-way ANOVA with Bonferroni correction. *P < 0.05; **P < 0.01; ***P < 0.001; ****P < 0.0001; ns, not significant. **J**–**L** Immunoprecipitation detection of total (**K**) and ubiquitinated (**L**) BACE1 levels in control and FBXW7 knockdown N2a cells. *P* values were determined by Student’s t test. **P < 0.01; ****P < 0.0001; ns, not significant. Data show mean ± S.D and each experiment was conducted with a minimum of three replicates.

FBXW7 serves as the substrate-recognition subunit within the SCF E3 ubiquitin ligase complexes. It mediates the ubiquitination and subsequent degradation of target proteins by specifically recognizes the conserved core sequence <RK > -S/T-P-P-X-S/T/E/D, commonly designated as the Cdc4 phosphodegron (CPD) [33–35]. <RK> indicates any amino acid except arginine (R) and lysine (K), while X indicates any amino acid. Alignment with previously reported FBXW7 recognition sequences revealed a potential CDP motif within the BACE1 protein, and this motif exhibited high conservation across mammals (Fig. 3A). Protein docking analyses revealed potential interactions between FBXW7 and the CPD motif region of BACE1, involving the following hydrogen bonds: GLU372 (FBXW7)–ARG122 (BACE1), ARG370 (FBXW7)–SER83 (BACE1), ARG363 (FBXW7)–GLN116 (BACE1), GLN306 (FBXW7)–THR87 (BACE1), GLN280 and GLU276 (FBXW7)–THR225 (BACE1), GLN273 and ARG226 (FBXW7)–GLU371 (BACE1), and HIS270 (FBXW7)–THR375 (BACE1) (Fig. 3B). Co-immunoprecipitation assays further confirmed the interaction between FBXW7 and BACE1 (Fig. 3C and Fig.S5A). Notably, residues mutation of the CDP sequence (G82R, S83G, P84G, P85G and T87A) in BACE1 abolished this interaction (Fig.S5B), validating the protein docking results (Fig. 3B).

To further investigate the effect of FBXW7 on BACE1 protein levels, we established N2a cell lines stably expressing flag-tagged wild type or mutant BACE1. Cycloheximide (CHX) chase assays showed that FBXW7 knockdown attenuated the degradation of BACE1-3Flag (Fig. 3D and E), indicating enhanced protein stability. To elucidate the pathway through which FBXW7 mediates BACE1 degradation, we applied inhibitors targeting the proteasomal or lysosomal pathways. Western blot analysis revealed that MG132 (a proteasome inhibitor) counteracted the upregulation of BACE1 induced by FBXW7 knockdown, whereas BafA1 (a lysosome inhibitor) did not, indicating that FBXW7 promotes BACE1 degradation via the proteasomal pathway (Fig. 3F–I). As previously described, overexpression of FBXW7a downregulates BACE1 protein levels (Fig. 2J); however, this effect was absent in cell lines expressing a CDP-mutated version of BACE1 (Fig. S4C and D). Since ubiquitination is essential for proteasomal degradation [36], we further examined the ubiquitination level of BACE1. FBXW7 knockdown significantly reduced BACE1 ubiquitination (Fig. 3J–L), but this effect was not observed with the CDP-mutated BACE1 (Fig.S4E-G). Collectively, these results suggest that FBXW7α specifically regulates the degradation of BACE1 through the ubiquitin-proteasome pathway.

### FBXW7 knockdown accelerates BACE1 transport to the Golgi apparatus and plasma membrane

**Fig. 4:**
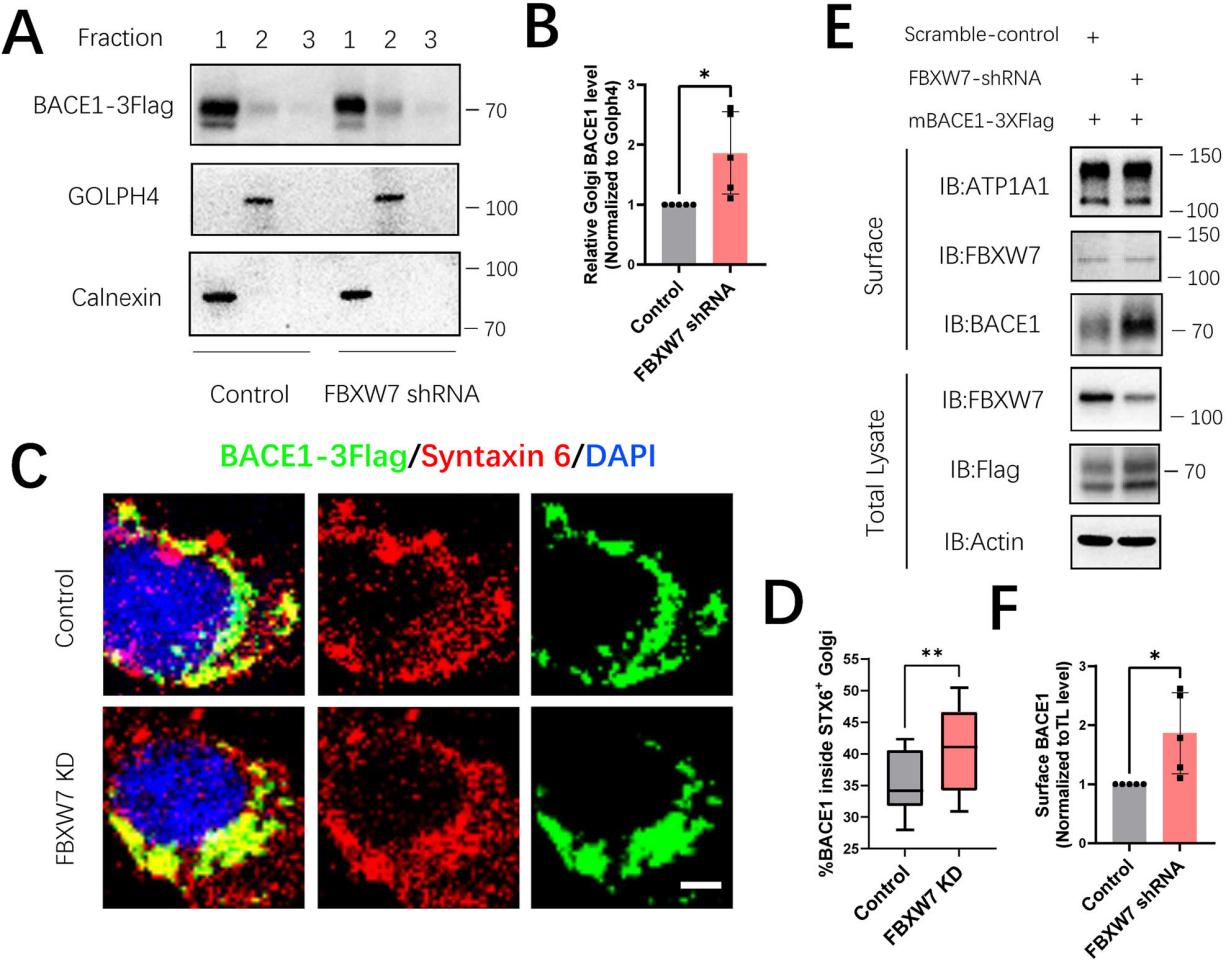
Deficiency in FBXW7α promotes BACE1 trafficking into the Golgi apparatus and enhances its membrane translocation. **A** Fractions extracted from control and FBXW7 knockdown cell lines overexpressing BACE1-3×Flag and then subjected to immunoblot analysis. GOLPH4 and Calnexin were used as Golgi and endoplasmic reticulum markers, respectively. **B** BACE1 trafficking to the Golgi apparatus was assessed by calculating the BACE1/GOLPH4 immunoblot intensity ratio; *n* = 4 in each group. Representative immunofluorescence images (**C**) and colocalization analysis **D** of BACE1-3×Flag with STX6-positive Golgi in N2a scramble control and FBXW7 knockdown stable cell lines overexpressing BACE1-3×Flag. Scale bar: 5 μm. *n* > 20 in each group. **E** Surface biotinylation assay was performed to detect the membrane translocation of BACE1-3×Flag in N2a cells upon FBXW7 knockdown (shRNA delivery through transient transfection). Na + /K+ ATPase(ATP1A1) was used as the plasma membrane marker. **F** BACE1 membrane translocation was assessed according to the Surface/Total(TL) BACE1 immunoblot intensity ratio. Data show mean ± S.D., and each experiment was conducted with a minimum of three replicates. *P* values were determined by Student’s t test. *P < 0.05; **P < 0.01.

The subcellular localization of BACE1 critically influences its activity and stability [37–39]. We isolated different cellular fractions and found that FBXW7 knockdown significantly increased the accumulation of BACE1 in the Golgi-enriched cellular fractions (Fig. 4A and B). Consistent with this, immunofluorescence results further confirmed that in FBXW7 stably knockdown N2a cells, the co-localization of BACE1 with Golgi marker (Syntaxin 6) was notably enhanced (Fig. 4C and D), suggesting the accumulation of BACE1 at the Golgi apparatus. To further verify the role of FBXW7 in regulating BACE1 trafficking, we performed biotin-labeled cell membrane isolation assays, which revealed that FBXW7 knockdown remarkably increased the distribution of BACE1 on the cell membrane (Fig. 4E and F). Biotin pull-down assay for membrane proteins revealed a very weak FBXW7α signal in the membrane fraction (detectable only under super-sensitive ECL conditions with prolonged exposure) (Fig. 4E). This minimal signal may have originated from indirect pull-down due to interactions between FBXW7α and certain membrane proteins, such as BACE1. However, whether FBXW7α or FBXW7β can actively localize to the cell membrane remains to be determined in future studies. Collectively, our results demonstrated that BACE1, as a ubiquitination substrate of FBXW7, was subject to FBXW7-mediated modulation of its intracellular trafficking.

### Restoration of FBXW7α expression ameliorates Aβ-related pathology and improves cognitive performance in 5×FAD mice

**Fig. 5:**
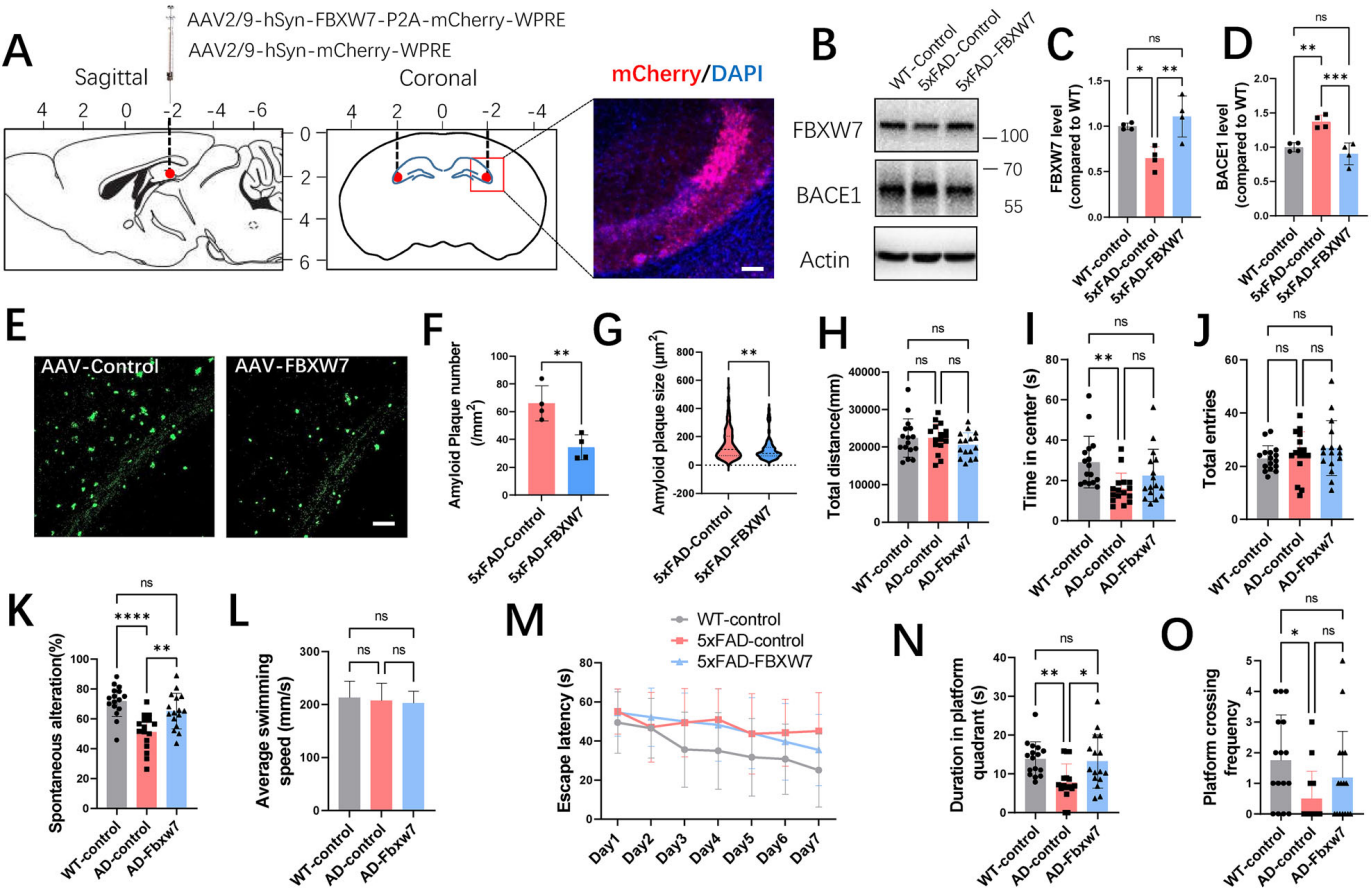
Restoration of FBXW7α levels in the hippocampus improves cognitive performance and reduce β-amyloid burden in 5×FAD mice. **A** Scheme showing the location for stereotactic injection of FBXW7α expressing or vector control AAV in the brain. Representative immunofluorescence image indicates mcherry expression in CA3 region of 5×FAD mice injected by vector control AAV for 4 weeks. Scale bar: 100 μm. Representative immunoblotting images **B** and quantitative analysis of FBXW7α **C** and BACE1 **D** expression in hippocampal tissues of WT and 5×FAD mice injected by control or FBXW7-expressing AAV for 12 weeks. Representative immunostaining **E** and quantification of amyloid plaques (**F**, **G**) with thioflavin S (ThioS) staining in the CA3 region of 5×FAD mice injected with AAVs carrying *FBXW7* transgene or control vector. Scale bar: 100 μm. Total locomotor activity **H** and center zone exploration **I** were recorded in open field test. Total arm entries **J** and spontaneous arm alterations **K** in Y-maze test. Training trial data, average swimming speed **L** and escape latency **M**, and platform crossing frequencies **N** and duration in target quadrant **O** in probe trial were recorded in the Morris water maze test. Data show mean ± S.D., and each experiment was conducted with a minimum of three replicates. **C**, **D**, **F**, **G**, *n* = 4 mice in each group. **H**–**O** n = 15 mice in each group. *P* values were determined by Student’s t test (**F**, **G**) or one-way ANOVA with Bonferroni correction (**C**, **D**, **H**–**O**). * *P* < 0.05; **P < 0.01; ***P < 0.001; ****P < 0.0001; ns, not significant.

Given that FBXW7 expression is downregulated in 5×FAD mice as early as before 6 months of age (relative to WT mice) (Fig. 1B and C), we constructed an FBXW7 expression vector driven by the *synapsin* gene promoter (hSyn), with hSyn-mCherry serving as the control. The vectors were packaged into adeno-associated viruses (AAVs), which were then bilaterally injected into the hippocampal regions of 4-month-old 5×FAD mice via stereotaxic surgery (Fig. 5A). 12 weeks later, we examined the protein expression in hippocampal tissues. The results showed that, in the FBXW7- restoration group (5×FAD-FBXW7), the protein level of FBXW7 was significantly upregulated compared with that in the AD control group (5×FAD-Control), and was close to that in the age-matched WT group (WT-Control). Concomitantly, the protein level of BACE1 was notably decreased following FBXW7 expression rescue (Fig. 5B–D). Additionally, we also detected the levels of APP C-terminal cleavage products and Aβ. It was found that FBXW7α rescue in 5×FAD mice significantly reduced the production of C99, Aβ_42_ and Aβ_40_ (Fig.S6A-E). Furthermore, thioflavin S staining confirmed that FBXW7α rescue significantly reduced Aβ accumulation in the hippocampal region (Fig. 5E–G). In addition, behavioral tests demonstrated that FBXW7α rescue had no effect on the total distance in the open field test (Fig. 5H), while it slightly alleviated the anxiety-like behavior of AD model mice (Fig. 5I). The Y-maze test (Fig. 5J and K) and Morris water maze tests (Fig. 5L–O) revealed that FBXW7α rescue remarkably improved the learning and memory abilities of 5×FAD mice. Together, our results demonstrated that restoring FBXW7α expression in AD model mice could alleviate Aβ-related pathology and improve cognitive functions.

## Discussion

Proteostasis refers to the cellular network that maintains protein homeostasis by coordinating protein synthesis, folding, trafficking, and degradation, preventing aggregation of misfolded proteins. Dysfunction in the proteasome pathway, a major protein degradation system, leads to impaired clearance of toxic protein aggregates such as Aβ and Tau in AD [40], α-synuclein in Parkinson’s disease [41], and Huntington (HTT) protein in Huntington’s disease [42], triggering synaptic dysfunction and neurodegeneration [43, 44]. Dysregulation of the ubiquitination-deubiquitination balance disrupts protein degradation, exacerbating proteostasis collapse and disease progression [45, 46]. As a typical E3 ubiquitin ligase, FBXW7 has been reported to regulate the degradation of MCL1, thereby influencing the survival of dopamine neurons [47]. Moreover, FBXW7 expression is reduced in the brains of AD patients [22, 28]. Integrated transcriptomic and proteomic analyses further identify FBXW7 as a central hub regulator involved in AD pathogenesis, with a particular role in modulating tau homeostasis [21]. Here, we confirmed that the α isoform of FBXW7 regulated APP processing and Aβ production by targeting BACE1 for ubiquitination and proteasomal degradation, suggesting that FBXW7α represents a potential therapeutic target for mitigating amyloid deposition in the AD brain.

The *FBXW7* gene expresses three conserved protein isoforms (defined as α, β, and γ), yet the differences in their tissue distribution and functional roles remain unresolved. Regarding tissue distribution, the FBXW7α isoform is widely expressed at high levels across various organs, establishing it as the major isoform of FBXW7 [24, 25]. In terms of subcellular localization, early studies identified FBXW7α in the nucleus, whereas FBXW7β was found to localize to the cytoplasm [26, 27]. However, a growing number of studies identified several cytoplasmic or membranal substrates of FBXW7α, such as DISC1[48], EGFR[14], TGF-β[49], and RIGI[50]. This finding suggested that FBXW7α is not exclusively confined to the nucleus and that its subcellular localization may exhibit cell type-specific patterns. In this study, our results originally demonstrated that FBXW7α was highly expressed in neuronal cells and exhibited dual localization within both the cytoplasmic and nuclear compartments (Fig.1 and S2). This finding not only provided advancing clues for investigating the function of FBXW7α in neural cells but also supported the possibility—proposed in other studies—that FBXW7α regulated the degradation of cytoplasmic proteins[14, 48–50].

Discriminating between the bands corresponding to FBXW7α and FBXW7β in Western blot assays has always been a persistent issue, and identification based solely on their theoretical molecular weights is unreliable. Our results also indicated that bands observed within the 50–70 kDa range—the predicted size for FBXW7β—actually correspond to FBXW7α (likely a cleavage product) or other unpredicted proteins rather than to FBXW7β itself (Fig. S1C-G). In contrast, exogenously expressed FBXW7β migrates at a position above 130 kDa (Fig. S1I and J). A panel of commercial antibodies used in this study exhibited their consistent specificity to FBXW7α isoform but not β. Therefore, at present we cannot determine the expression pattern of FBXW7β. Furthermore, tag-labeled FBXW7β exhibited slow migration rate inconsistent with its predicted molecular weight (Fig. S1I and J). This discrepancy may be due to involvement of FBXW7β in forming high-molecular-weight protein complexes by forming homo-oligomers or complexes with other proteins. The differential localization of FBXW7α and FBXW7β suggests that they may have distinct biological functions (Fig.S2A). This is confirmed by our finding that FBXW7α specifically regulates the degradation of BACE1 in this study. Collectively, the expression pattern and biological function of FBXW7β remain to be elucidated, and developing an antibody that specifically recognizes FBXW7β is key to future investigations.

**Fig. 6:**
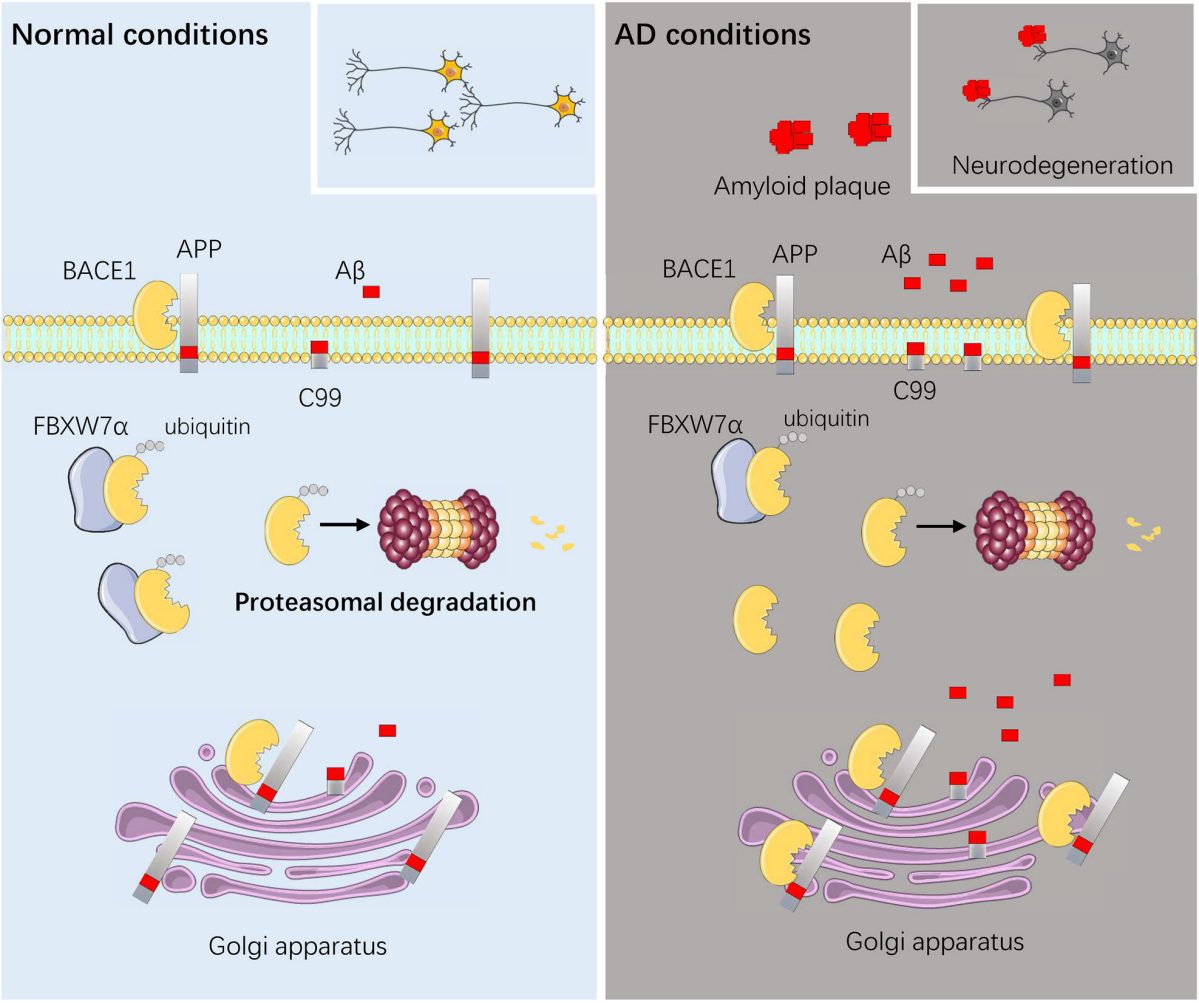
Schematic showing the mechanisms of FBXW7 in modulating amyloid pathology by targeting BACE1 degradation.

In the brains of AD patients, the expression level of BACE1 is significantly upregulated, particularly in neurons adjacent to neuritic plaques [51]. Dysfunction of the ubiquitin-proteasome system contributes significantly to the accumulation of BACE1 in AD brains. BACE1 can be ubiquitinated and subsequently degraded via the proteasome pathway [52–55]. We provided advancing evidence that FBXW7α interacted with BACE1 and mediated the degradation of BACE1 via the ubiquitin-proteasome pathway (Figs. 3 and S5). Moreover, FBXW7 also appeared to regulate BACE1 subcellular distribution (Fig. 4). BACE1 exhibits a predominant subcellular localization in endosomes and the trans-Golgi network, which provides the low pH microenvironment of for the optimal cleavage activity of BACE1 [56]. Therefore, the increased trafficking of BACE1 to the Golgi apparatus caused by FBXW7 deficiency ultimately augments the β-cleavage of APP and the resultant Aβ production (Figs. 5 and S6). In addition, restoration of FBXW7α expression in the hippocampi tissue of 5×FAD mice ameliorated Aβ burden and improved cognitive function, further proving the significant role of FBXW7α in alleviating AD pathologies (Fig. 5). The mechanism diagram is shown in Fig. 6.

Numerous E3 ligases have been investigated as AD therapeutic targets, acting on two core pathological cascades: tau hyperphosphorylation and Aβ generation. The E3 ubiquitin ligase Praja1 (Praja ring finger ubiquitin ligase 1) directly counteracts tau pathology by promoting the poly-ubiquitination and proteasomal degradation of tau [57]. F-box and leucine rich repeat protein2 (FBL2) regulates APP metabolism by promoting ubiquitination-dependent APP degradation [58]. F-Box Protein 2 (FBX2) mediates degradation of BACE1 and attenuates AD-associated amyloidosis [55]. However, two critical limitations restrict the clinical translation of most E3 ligase-targeted AD therapies. First, the majority of these E3 ligases lack strict substrate specificity, targeting multiple endogenous proteins beyond AD-related pathogenic factors, resulting in a high risk of off-target effects and systemic toxicity. Second, the development of small-molecule activators for most E3 ligases remains technically challenging. Therapeutic strategies for AD generally require E3 ligase activation to accelerate pathogenic protein clearance, but most reported E3 modulators are inhibitors, and the rational design of selective activators is hindered by the complex structural dynamics of E3 ligase complexes. The FBXW7-mediated BACE1 ubiquitination and degradation pathway potentially exhibits distinct advantages over other E3 ligase targets for AD. First, FBXW7 has well-defined substrate specificity, recognizing substrates via conserved phosphodegron motifs, which markedly minimizes off-target risks compared to other E3 ligases such as Praja1, FBL2 and FBX2. Second, unlike most E3 ligases with limited clinically translatable modulators, FBXW7 activity can be upregulated by sorafenib, an FDA-approved oral multikinase inhibitor for the treatment of unresectable hepatocellular carcinoma. Sorafenib could enhance FBXW7 expression and attenuate cognitive deficits and tau pathologies in PS19 tauopathy model mice [23]. This drug repurposing potential significantly accelerates clinical translation, bypassing the lengthy and costly de novo drug development process. Furthermore, FBXW7 has been shown to modulate both tau and Aβ pathology, presenting promising therapeutic potential for patients at various stages of AD.

## Conclusions

In summary, our findings demonstrated that the E3 ligase FBXW7α contributes to AD-related amyloid pathology by regulating the degradation and translocation of BACE1, providing a potential therapeutic target for AD treatment.

## Materials and methods

### Animal studies

The C57BL/6 J wild type and 5×FAD transgenic mice [Tg(APPSwFlLon, PSEN1*M146L*L286V)6799Vas] were purchased from the Shanghai Model Organisms Center, Inc, and bred in Laboratory Animal Center of Jining Medical University. The mice were housed under specific pathogen-free (SPF) conditions with controlled environmental parameters: temperature maintained at 22-26°C, relative humidity at 40–60%, and a 12 h light/dark cycle. Animals had ad libitum access to standard rodent chow and filtered water. Male mice of various monthly ages were utilized in this study.

### Cell culture and reagents

Mycoplasma-free (detected by Mycoplasma Detector Kit, D101-01, Vazyme) mouse Neuro2a cells (CCL-131 ATCC) and human embryonic kidney–293 cells (HEK293, CRL-1573 ATCC) were cultured in Dulbecco’s modified Eagle’s medium (DMEM; Gibco, Thermo Fisher Scientific, Waltham, MA, USA) supplemented with 10% (v/v) FBS (Gibco) and 1% (v/v) antibiotics (100 unit/mL penicillin, 100 μg/mL streptomycin). Neuro2a cells stably overexpressing human APP Swedish mutant (APPswe, K670N and M671L), mouse BACE1-3×Flag, mouse BACE1-3×Flag mutant (G82R, S83G, P84G, P85G and T87A), and FBXW7 shRNA cassettes were obtained by transfection with corresponding lentiviral vectors and screened by puromycin (5 μg/ml). All cells were maintained at 37 °C and 5% CO_2_. Cycloheximide (MedChemExpress, HY-12320) at the concentration of 100 μg/mL was used to block protein synthesis. MG132 (MedChemExpress, HY-13259) and Bafilomycin A1 (Bafa1; MedChemExpress, HY-100558) were used to block proteasome- and lysosome- mediated protein degradation, respectively.

### Plasmids and transfection

To construct the *Fbxw7* knockdown plasmid, sense and antisense fragments targeting CAGCACAGAATTGATACAAAC were synthesized and cloned into the sites of AgeI and EcoRI in the pLKO.1-puro plasmid and verified by Sanger sequencing. To construct the FBXW7 or BACE1 overexpression plasmids, corresponding genes coding sequences were amplified and cloned into pcDNA4 or pLVX-Puro vectors. Flag tag fusion and amino acids mutant were performed by overlapping PCR. The transfection procedure was conducted using EndoFectin Max transfection reagent (iGenebio, EF013) according to the guidelines provided by the manufacturer.

### RNA extraction and RT-qPCR analysis

Total RNA was extracted from mouse brain tissues and cells using TRIzol reagent (Invitrogen, Grand Island, NY, USA) according to the manufacturer’s instructions. cDNA was synthesized from 1 μg of total RNA using the Strand cDNA Synthesis Kit (D7168M, Beyotime). Quantitative real-time PCR (RT-qPCR) was performed using SYBR qPCR Master Mix (Q711-02, Vazyme, China) on an Applied Biosystems 7500 Real-Time PCR System (Applied Biosystems). Relative mRNA expression levels were calculated using the ΔΔCT method with *Gapdh* as the internal reference control. The primer sequences used in this study are listed in Supplementary Table 1.

### Protein extraction and western blot

To evaluate the effects of different treatments on the levels of target proteins, total protein lysates were extracted from mouse brain tissues or cultured cells using RIPA buffer (P0013B, Beyotime, China) and subsequently analyzed by western blotting. Protein samples were separated by electrophoresis on 8–15% Tris-glycine sodium dodecyl sulfate-polyacrylamide (SDS-PAGE) gels, with tricine SDS-PAGE gels being specifically employed for the detection of APP cleavage products C99 and C83 [59]. Following electrophoresis, the separated proteins were transferred onto PVDF membranes (IPVH00010, Millipore; E801, Vazyme). For blocking, the membranes were incubated with 5% non-fat dry milk prepared in TBST (Tris-Buffered Saline containing 0.1% Tween-20) for 1 hour at room temperature. The membranes were probed with the indicated antibodies: anti-APP (25524-1-AP, Proteintech), anti-BACE1 (#5606, CST), anti-APP-CTF (A8717, Sigma-Aldrich), anti-FBXW7 (28424-1-AP, Proteintech; ab192328, ab12292 Abcam; 40-1500, Invitrogen; sc-293423, Santa Cruz; A301-721, Bethyl), anti-Flag (20543-1-AP and 66008-4-Ig, Proteintech), anti-ubiquitin (10201-2-AP, Proteintech; sc-8017, Santa Cruz), anti-GOLPH4 (31083-1-AP, Proteintech), anti-Calnexin (66903-1-Ig, Proteintech), anti-ATP1A1 (14418-1-AP, Proteintech) and β-Actin (ZSGB-BIO, AC-15). HRP-labeled secondary antibodies and electrochemiluminescence substrate (WBKLS0100, Milipore; E423, Vazyme) were used for visualization. The expression levels were quantitatively assessed by measuring the grayscale intensity of each band and subsequently analyzed using ImageJ software.

### Immunoprecipitation

Total protein lysates were extracted from cells using RIPA buffer (P0013D, Beyotime) as previously described. For immunoprecipitation, cell lysates were incubated overnight at 4 °C with either target-specific primary antibodies or control IgG (A7016, Beyotime). Subsequently, protein A/G beads (P2055, Beyotime) were added, and the mixture was incubated for an additional 2 h at 4 °C with gentle rotation. The immune-complexes were then washed three times with RIPA lysis buffer (10 min per wash, 30 min total) to remove nonspecific binding. After the final wash, the precipitated proteins were eluted and subjected to Western blot analysis. To detect endogenous ubiquitination level of FBXW7 in mouse brains, rabbit FBXW7 antibody (28424-1-AP, Proteintech) was used for immunoprecipitation, and mouse ubiquitin antibody (sc-8017, Santa Cruz, Proteintech) was used for immunoblotting (Fig. 1F). To detect ubiquitination level of exogenous BACE1-3Flag in cells, mouse Flag antibody (66008-4-Ig, Proteintech) was used for immunoprecipitation, and rabbit ubiquitin antibodies were used for immunoblotting (Fig. 3J and Fig.S4G).

### Immunofluorescence

Immunofluorescence staining was performed following an established protocol [60]. Briefly, cells were cultured on glass coverslips, fixed with 4% paraformaldehyde (PFA) for 20 min at room temperature (RT). For brain slice staining, PBS-perfused mouse brains were dissected and fixed in 4% paraformaldehyde (PFA) for one week at 4°C, followed by sectioning using cryostat microtome. Cells or tissue slices were permeabilized with 0.3% Triton X-100 in PBS. Non-specific binding was blocked by incubation with 10% normal goat serum for 1 h at RT. The tissue slices or cells were incubated with the following primary antibodies overnight at 4°C: rabbit anti-FBXW7 (28424-1-AP, Proteintech), mouse anti-NeuN (66836-1-Ig, Proteintech), mouse anti-Aβ (6E10, BioLegend), mouse anti-Syntaxin 6 (60059-1-Ig, Proteintech), and anti-Flag (20543-1-AP, Proteintech). After PBS washes, samples were incubated for 2 h at RT with species-matched secondary antibodies conjugated to Alexa Fluor 488 or 568 (1:500; Thermo Fisher Scientific). Nuclei were counterstained with DAPI (Beyotime) for 10 min, and coverslips were mounted onto slides using anti-fade mounting medium. The images were captured by a confocal microscope (Zeiss LSM 980), and quantification of immunostaining was performed using ImageJ software.

### Enzyme-linked immunosorbent assay (ELISA)

Brain tissue was homogenized in ice-cold TBSTx buffer (TBS containing 1% Triton X-100) supplemented with protease and phosphatase inhibitors. The homogenates were centrifuged at 12,000 rpm for 30 min at 4 °C, and the resulting supernatant was collected for analysis of detergent-soluble Aβ. The pellet was subsequently solubilized in 5 M guanidine hydrochloride (GND) to extract detergent-insoluble Aβ aggregates. Prior to ELISA analysis, all samples were diluted 1:20 in appropriate buffer. Aβ_1-40_ and Aβ_1-42_ levels were quantified using commercial ELISA kits (Invitrogen, USA) following the manufacturer’s recommended protocol.

### Cell surface biotinylation assay

The biotinylation assay was performed as previously described [46]. Briefly, cells were washed three times with phosphate-buffered saline (PBS) followed by incubation with 1 mg/mL EZ-Link Sulfo-NHS-LC-Biotin (HY-D0799, MedChemExpress) in PBS at 4°C for 30 min with gentle shaking. To quench the reaction, cells were washed twice with ice-cold PBS containing 1 M glycine. After an additional PBS wash, cells were lysed in lysis buffer (1% NP-40, 0.9% NaCl, 10 mM Tris-HCl, pH 7.4) supplemented with protease inhibitor cocktail. The cell lysate was then incubated with pre-washed streptavidin agarose beads (P2159, Beyotime) overnight at 4 °C with constant orbital shaking. Following incubation, the beads were washed three times with PBS, and bound proteins were eluted by boiling in 2×SDS loading buffer for 10 min. The eluted proteins were subsequently analyzed by immunoblotting.

### Subcellular fractionation

Subcellular fractionation was performed as previously described [61] using the Golgi Apparatus Enrichment Kit Stain Kit (GO-037, invent).

### Adeno-associated virus (AAV) packaging

The mouse *Fbxw7α* coding sequence was cloned into the pAAV-hSyn-FBXW7-P2A-mcherry-WPRE plasmid (Obio Technology, Shanghai) and confirmed by sequencing. The human *Synapsin1* promoter (hSyn) in this construct drives neuron-specific expression of the downstream genes. The pAAV-hSyn-mcherry-WPRE plasmid served as the control. Subsequently, the recombinant plasmids were packaged into AAV2/9 viral particles by Obio Technology. The viral titers exceeded 2.0E + 13 v.g./ml.

### Stereotaxic injection of AAVs into the brain

Wild-type and 5×FAD mice were used for AAVs injection. 5×FAD mice were randomly divided into two groups: 5×FAD + AAV-control and 5×FAD + AAV-FBXW7 expression. Mice were anesthetized using a RWD anesthesia machine (RWD Life Science) with 1–2% isoflurane to maintain a stable anesthetic state throughout the operation. The anesthetized animal was fixed on a stereotaxic frame to ensure the skull was aligned with the bregma as the reference point. A Foredom electric drill (Foredom Electric Co.) equipped with a sterile drill bit (diameter: 0.5 mm) was used to drill a small burr hole at the target coordinate. A 2.5 µL microliter syringe (7632-01, Hamilton) was loaded with the AAVs solution, and the syringe was mounted on the stereotaxic frame’s injection arm. The coordinates used for bilaterally hippocampus injections were anteroposterior, −2.0 mm; mediolateral, ± 2.0 mm; dorsoventral, −2.0 mm. After confirming the needle reached the target depth, AAVs (1.0e^9^ GC) were injected at a constant speed of 100 nL/min using a syringe pump (Harvard Apparatus). Following the completion of injection, the needle was retained for an additional 10 min to allow full diffusion of the virus. The scalp incision was sutured with sterile absorbable sutures, and the wound surface was disinfected with povidone-iodine. The animal was placed in a warm recovery cage until full awakening from anesthesia, then returned to its home cage for subsequent feeding and experimental observation.

### Open field test (OFT)

The Open Field Test was employed to assess autonomous behaviors, locomotor activity, and anxiety levels of mice in a novel environment. The testing apparatus consisted of a topless rectangular arena (60 cm × 60 cm × 40 cm), with a defined central zone measuring 30 cm × 30 cm. Following established protocols [62], each mouse was gently placed in one corner of the chamber and allowed to freely explore for 6 min. Behavioral parameters were automatically recorded using an infrared tracking system throughout the test session.

### Y Maze test

The Y-maze test was employed to assess the impact of FBXW7-AAV injection on spatial learning and memory in mice. The apparatus consists of a central equilateral triangular chamber connected to three identical radial arms (labeled A, B, and C) positioned at 120° angles relative to each other. Distinct visual cues were placed at the distal end of each arm to facilitate spatial orientation. Following established protocols with minor modifications [31], the experimental procedure was conducted as follows: Mice were initially placed in the designated start arm (arm A) and permitted to freely explore all three arms for a 6-minute session. Their movements were automatically tracked using video-based analysis software, which recorded both the total number of arm entries and correct spontaneous alternations (defined as spontaneous entries into all three different arms without repetition). This alternation behavior serves as an indicator of spatial working memory.

### Morris water maze (MWM) test

The Morris Water Maze (MWM) test was employed to assess spatial learning and memory in mice. MWM test was performed as previously described [62]. The MWM apparatus consisted of a circular pool filled with opaque water, surrounded by walls displaying four distinct visual cues. During the acquisition phase (Stage 1, Day1-2), each session began by placing the mouse in the diagonal quadrant opposite to a visible escape platform, allowing 60 s for exploration. Swimming speed and escape latency (defined as the time from water entry to platform mounting) were recorded. Subsequently, in the hidden platform phase (Stage 2, Day3-7), the platform was submerged below the water surface while maintaining the same spatial location. Escape latencies were measured to evaluate spatial learning. During days 1–7, mice were released from two distinct starting points, each used once per day. For the final probe trial (Stage 3, Day 8), the platform was removed, and mice were reintroduced into the pool from the original starting position. Their exploratory behavior was monitored for 60 s, during which the time spent in each quadrant and the number of platform-area crossings were recorded as measures of spatial memory retention.

### 4D-Label-free proteomics

Sample lysis and protein extraction were performed using SDT buffer (4% SDS, 100 mM Tris-HCl, pH 7.6). Protein concentration was quantified using the BCA Protein Assay Kit (Bio-Rad, USA). For each sample, 20 µg of protein was mixed with 5×loading buffer, denatured by boiling for 5 min, and resolved on a 4–20% SDS-PAGE gel at a constant voltage of 180 V for 45 min. Protein bands were visualized by Coomassie Brilliant Blue R-250 staining. Protein digestion was carried out using trypsin following the filter-aided sample preparation (FASP) method as described by Matthias Mann. Briefly, excised gel bands were destained, reduced, alkylated, and digested overnight at 37 °C. The resulting peptides were desalted using C18 solid-phase extraction cartridges (Empore™ SPE Cartridges C18, standard density; 7 mm bed I.D., 3 ml volume; Sigma). After desalting, peptides were concentrated by vacuum centrifugation and reconstituted in 40 µL of 0.1% (v/v) formic acid for downstream analysis.

LC-MS/MS was used for peptides detection and quantification. LC-MS/MS analysis was performed on a timsTOF Pro mass spectrometer (Bruker) coupled to a Nanoelute system (Bruker). Peptides were separated on a C18 column (25 cm × 75 μm ID, 1.9 μm; Thermo Scientific) using a 300 nL/min gradient from 95% buffer A (0.1% FA in water) to buffer B (99.9% ACN/0.1% FA). The MS was operated in positive ion mode with a 1.5 kV electrospray voltage and a 100–1700 m/z scan range. The MS raw data for each sample were combined and searched using the MaxQuant 1.6.14 software and Uniprot database for identification and quantitation analysis.

Protein domains were identified using InterProScan with the Pfam database. Annotated proteins were then BLAST-searched against the KEGG database (http://geneontology.org/) to obtain KEGG orthology (KO) identifiers and pathway mappings. Functional enrichment analysis was performed using Fisher’s exact test, with the full quantified proteome as the background. P-values were adjusted via the Benjamini-Hochberg method, and only terms with FDR < 0.05 were considered significant.

### Statistical analysis

All data are expressed as mean ± standard deviation (s.d.). The sample size (*n*) for each experimental group is indicated in the figure legends and represents independent biological replicates. For in vitro experiments, each assay was independently repeated at least three times using different biological samples. For animal experiments, each group contained at least 14 (for behavior tests) and 4 (for pathology-associated detection) mice, as indicated in the figure legends. The sample size was determined based on previous studies in the field and established laboratory experience with analogous models, consistent with common practices in the discipline.

No data points were excluded from the final dataset during the analysis. No formal randomization procedure was employed for sample or animal assignment in this study, while group allocation was conducted in a non-biased manner. Formal blinding was not implemented during experimental procedures or outcome evaluation. However, to mitigate potential bias, data analysis was conducted by researchers who were not involved in the administration of treatments.

Statistical significance was assessed using appropriate tests (paired t-tests, one-way ANOVA, or two-way ANOVA) based on experimental design. Normal distribution and variance homogeneity were assessed by Prism’s in-built algorithms. *P* < 0.05 was considered statistically significant. All statistical analyses and data visualization were conducted using GraphPad Prism 9 software (GraphPad Software, USA).

## Supplementary information

Raw data-Western blot Images

Supplement Figures and captions

Supplement table

## Data Availability

The data that support the findings of this study are available on request from the corresponding author.
