# Supplementary material for: FBXW7α regulates amyloid pathology by mediating ubiquitination and degradation of BACE1 in Alzheimer’s disease
Source: Cell Death Discov. 2026 May 20;12:300. doi: 10.1038/s41420-026-03159-y (PMC13358112; doi:10.1038/s41420-026-03159-y)
Supplement: Supplementary file 1 — Raw data-Western blot Images [file 41420_2026_3159_MOESM1_ESM.pdf]

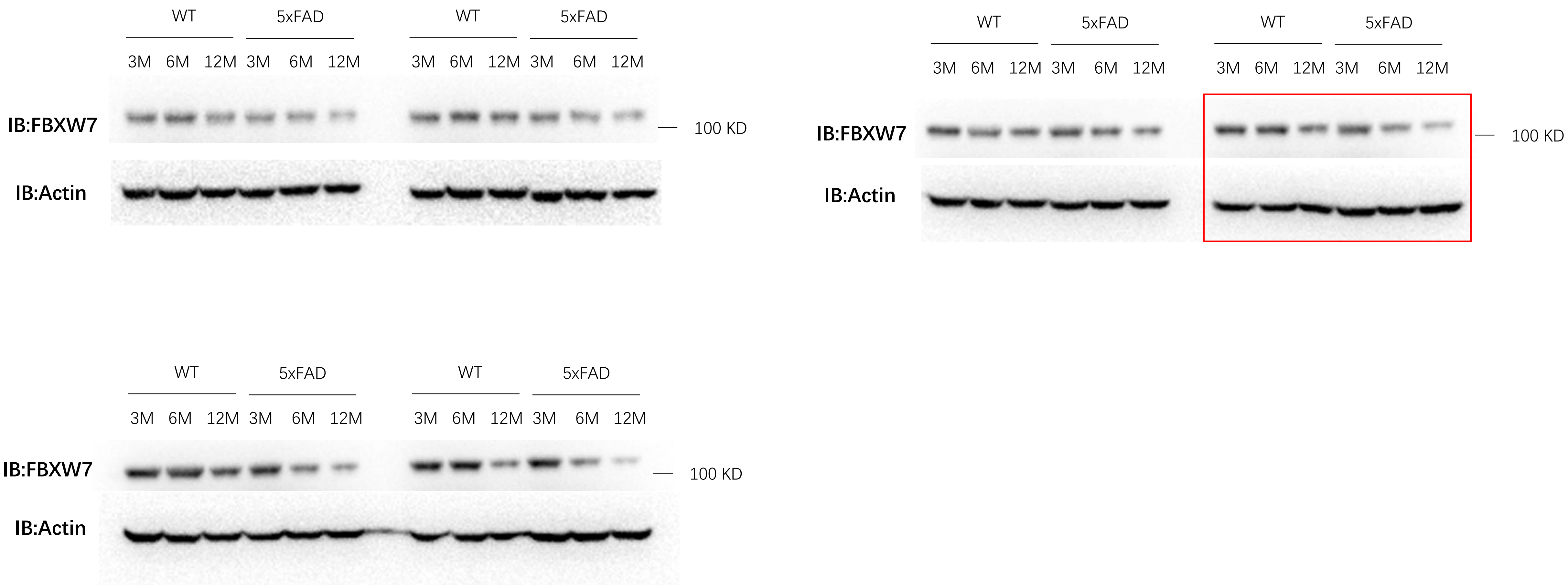

Cortex

Figure 1D

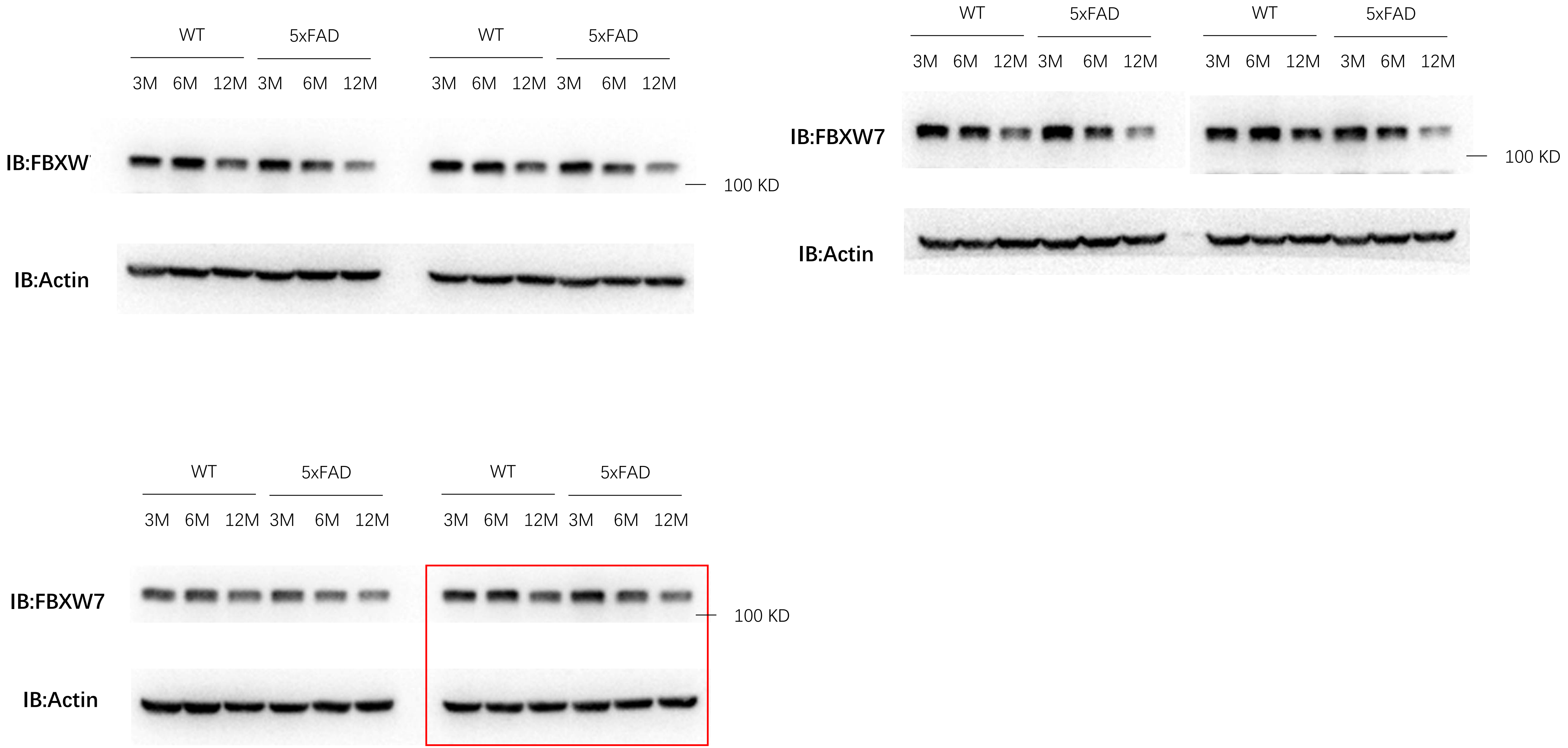

Hippocampus

Figure 1D

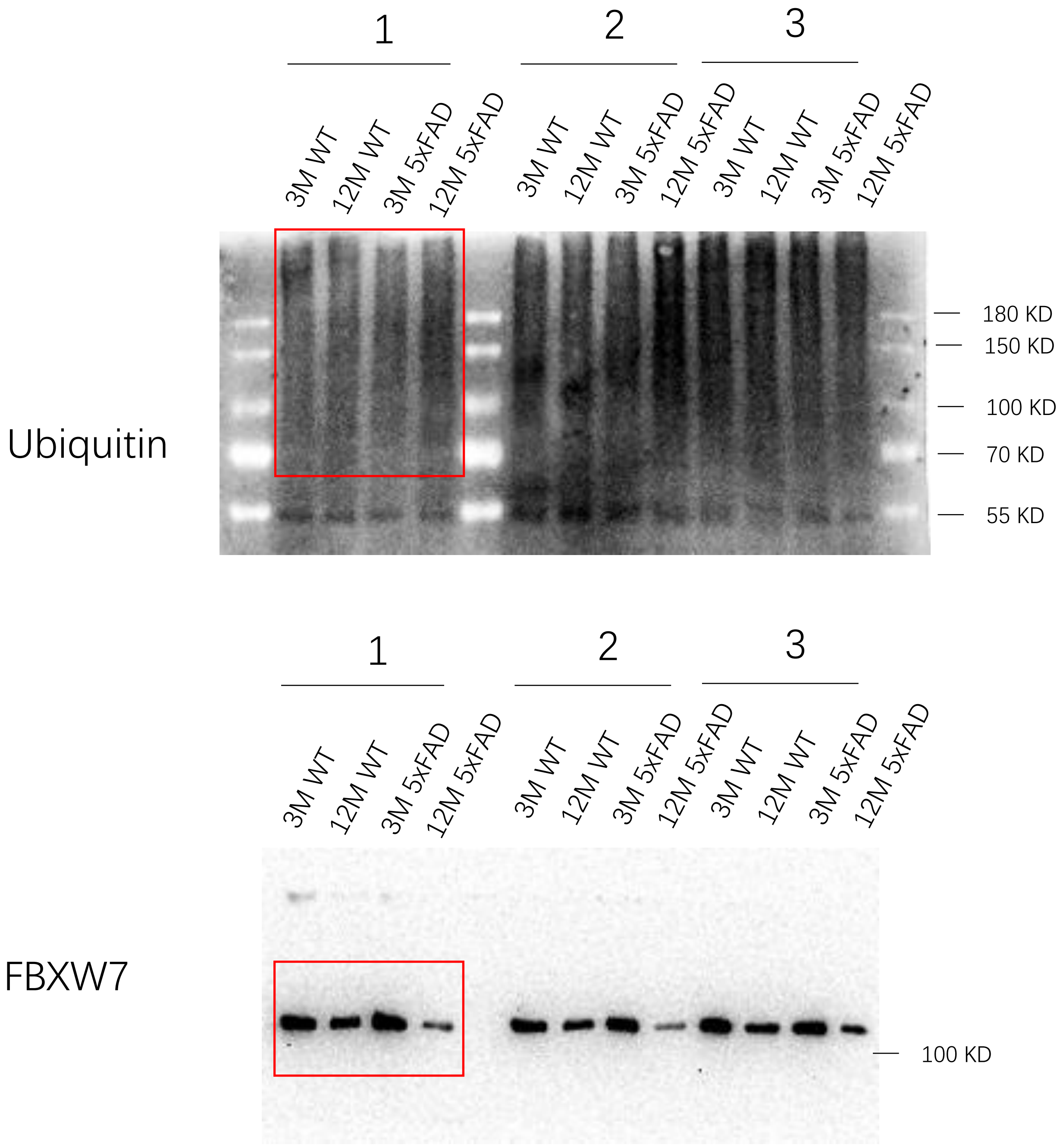
1
2
3
3M WT
12M WT
3M 5xFAD
12M 5xFAD
Ubiquitin
180 KD
150 KD
100 KD
70 KD
55 KD
FBXW7
100 KD

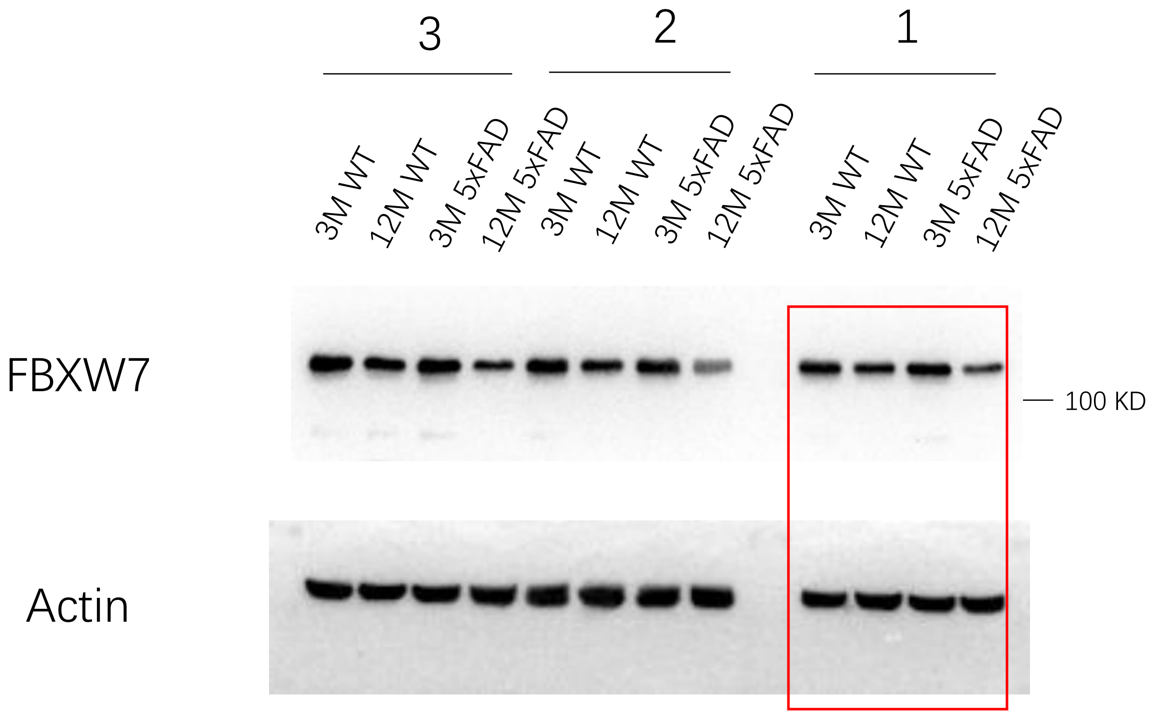
3
2
1
3M WT
12M WT
3M 5xFAD
12M 5xFAD
FBXW7
100 KD
Actin

Figure 1F

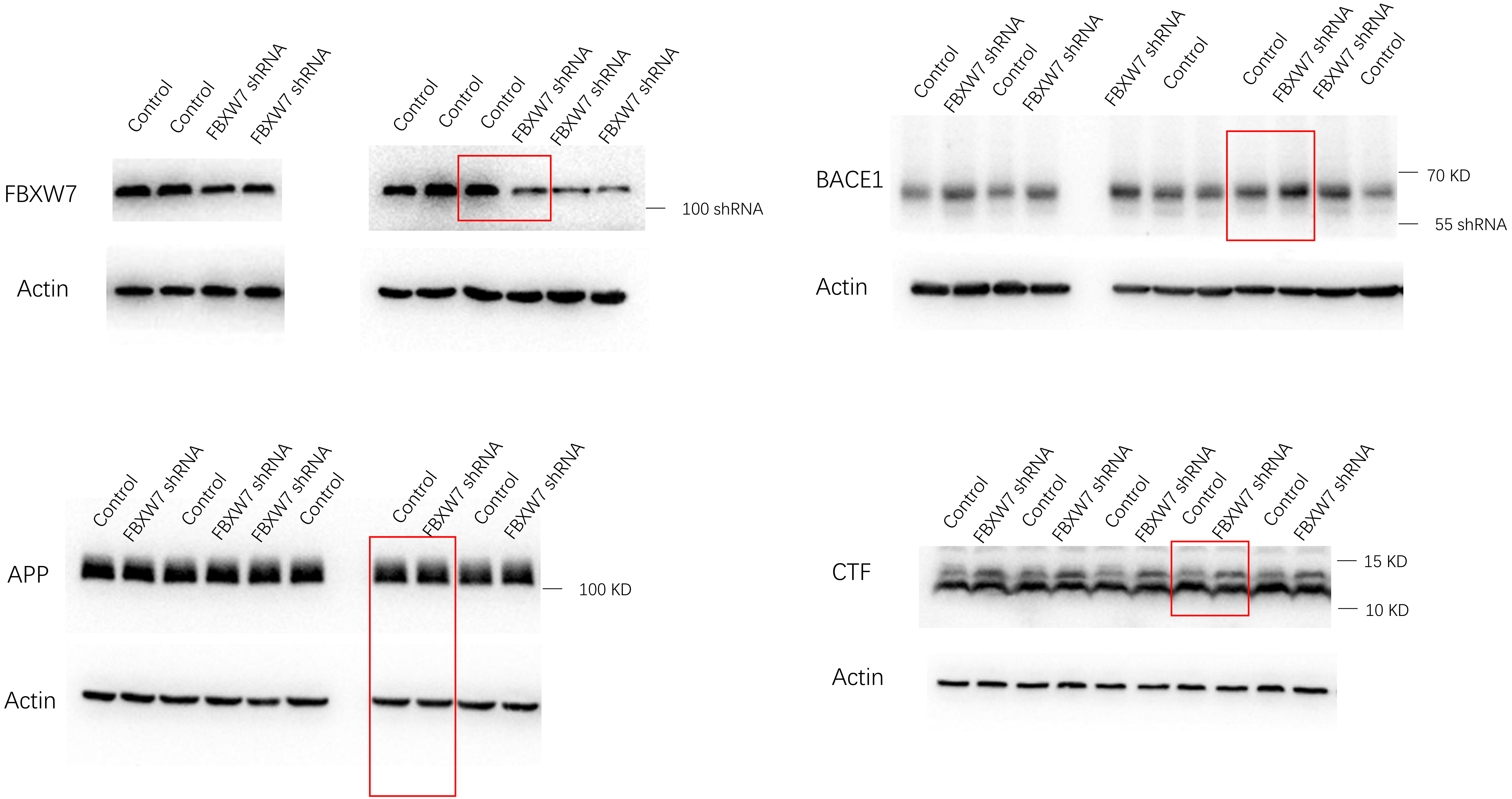
Control
Control
FBXW7 shRNA
FBXW7 shRNA
Control
Control
Control
FBXW7 shRNA
FBXW7 shRNA
FBXW7 shRNA
FBXW7
Actin
100 shRNA
Control
FBXW7 shRNA
Control
FBXW7 shRNA
FBXW7 shRNA
Control
Control
FBXW7 shRNA
FBXW7 shRNA
Control
BACE1
Actin
70 KD
55 shRNA
Control
FBXW7 shRNA
Control
FBXW7 shRNA
FBXW7 shRNA
Control
Control
FBXW7 shRNA
Control
FBXW7 shRNA
APP
Actin
100 KD
Control
FBXW7 shRNA
Control
FBXW7 shRNA
Control
FBXW7 shRNA
Control
FBXW7 shRNA
Control
FBXW7 shRNA
CTF
Actin
15 KD
10 KD

Figure 2A

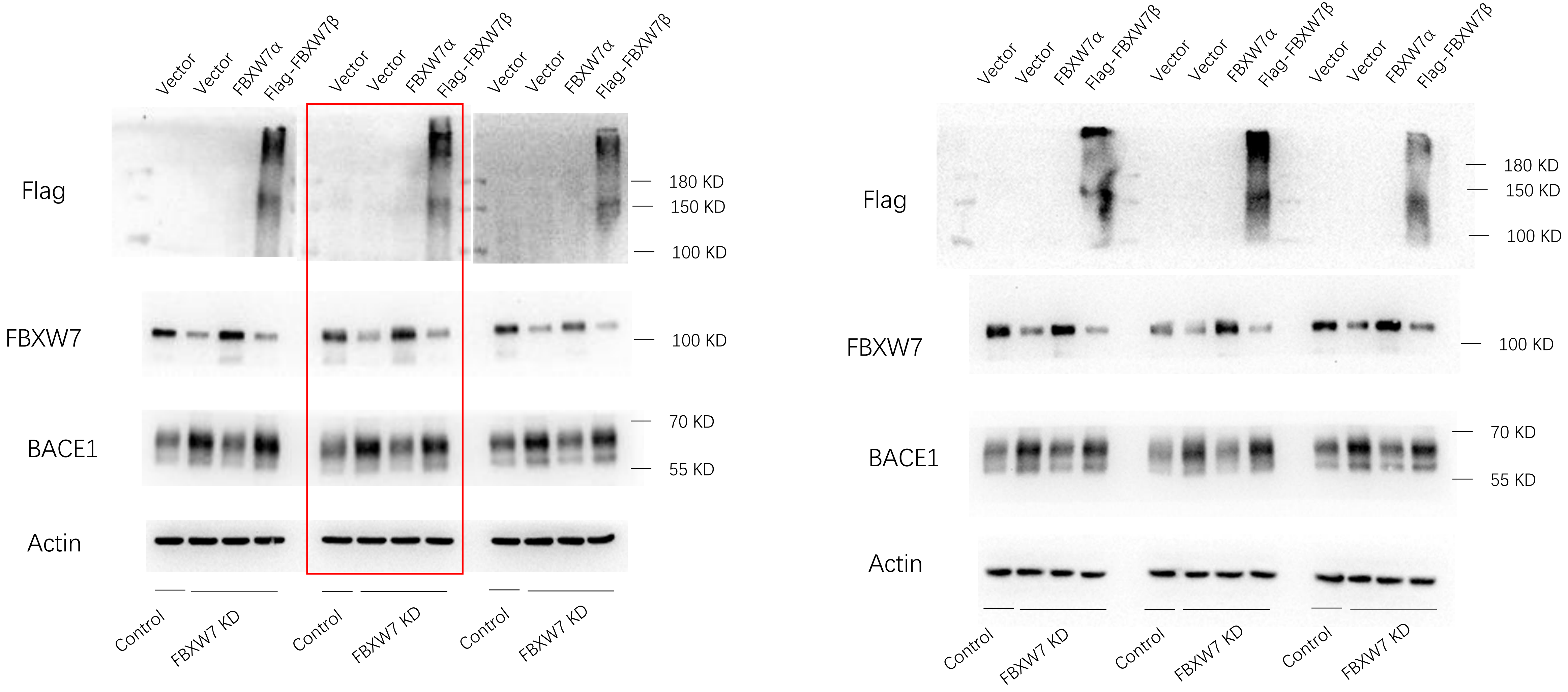

Vector
Vector
FBXW7α
Flag-FBXW7β
Flag
FBXW7
BACE1
Actin
180 KD
150 KD
100 KD
70 KD
55 KD
Control
FBXW7 KD

Figure 2G

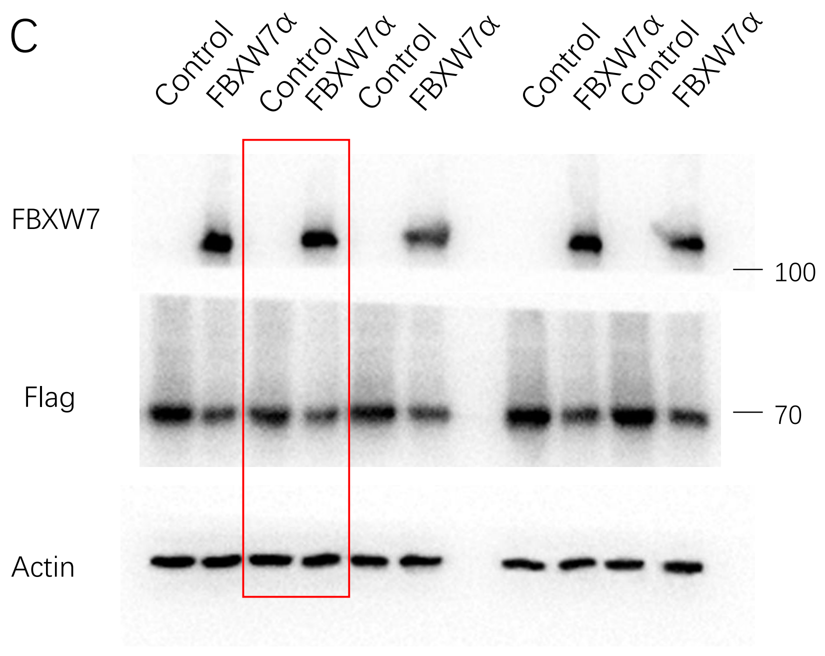

Figure 2J

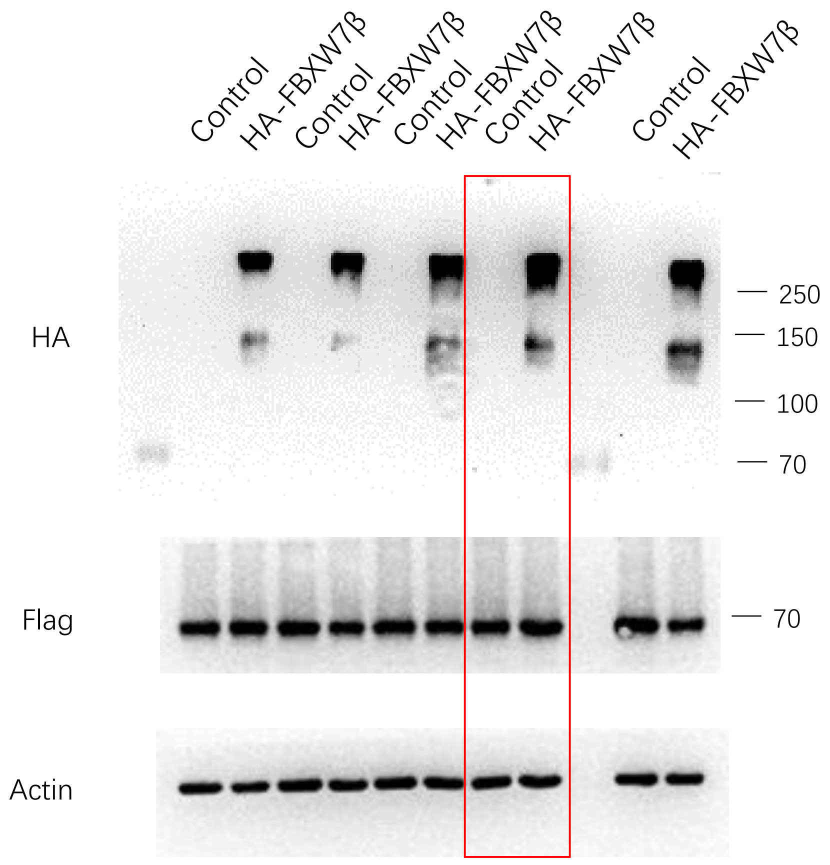

Figure 2K

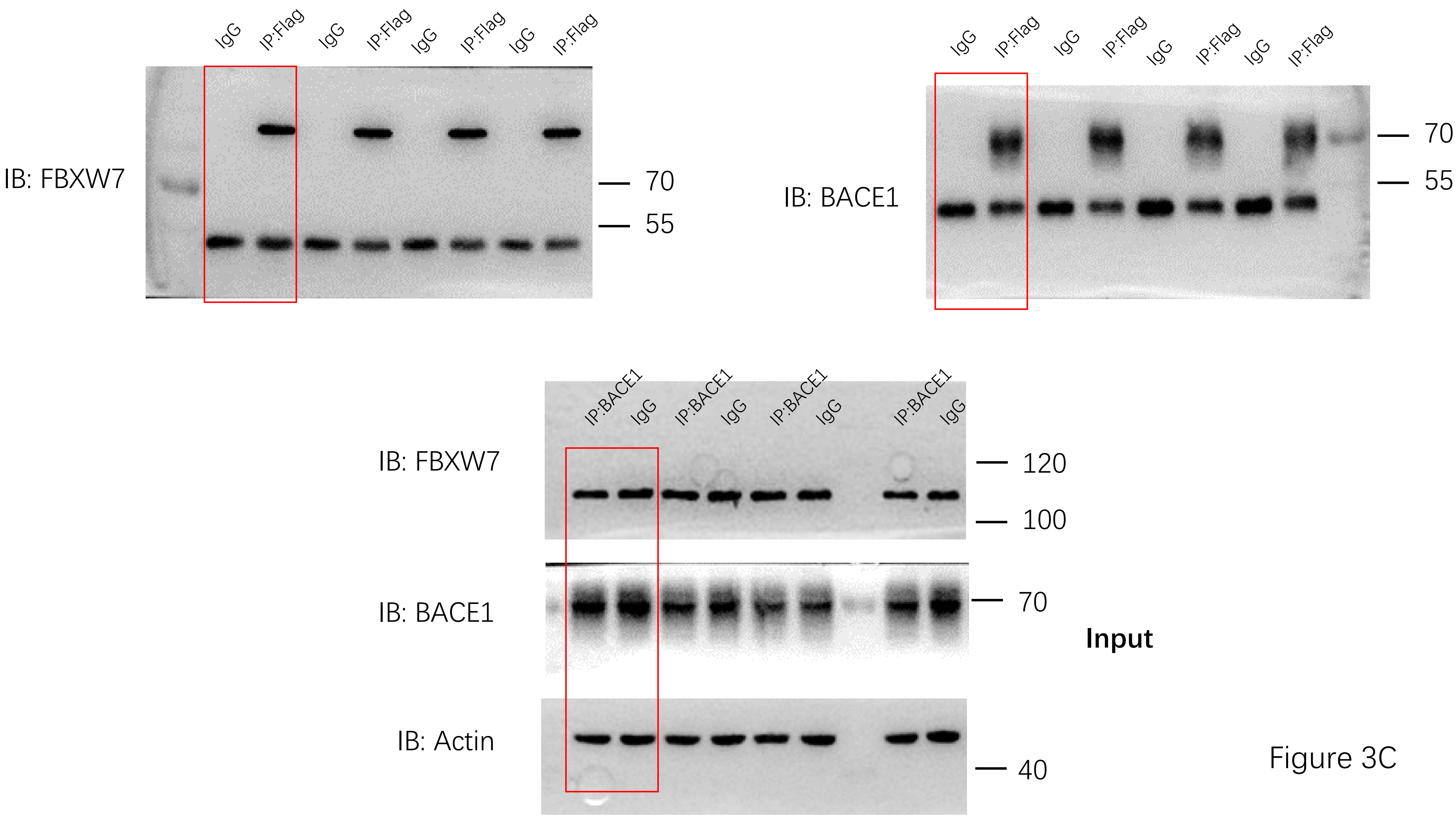
IgG
IP:Flag
IgG
IP:Flag
IgG
IP:Flag
IgG
IP:Flag
IB: FBXW7
70
55
IgG
IP:Flag
IgG
IP:Flag
IgG
IP:Flag
IgG
IP:Flag
IB: BACE1
70
55
IP:BACE1
IgG
IP:BACE1
IgG
IP:BACE1
IgG
IP:BACE1
IgG
IB: FBXW7
120
100
IB: BACE1
70
Input
IB: Actin
40

Figure 3C

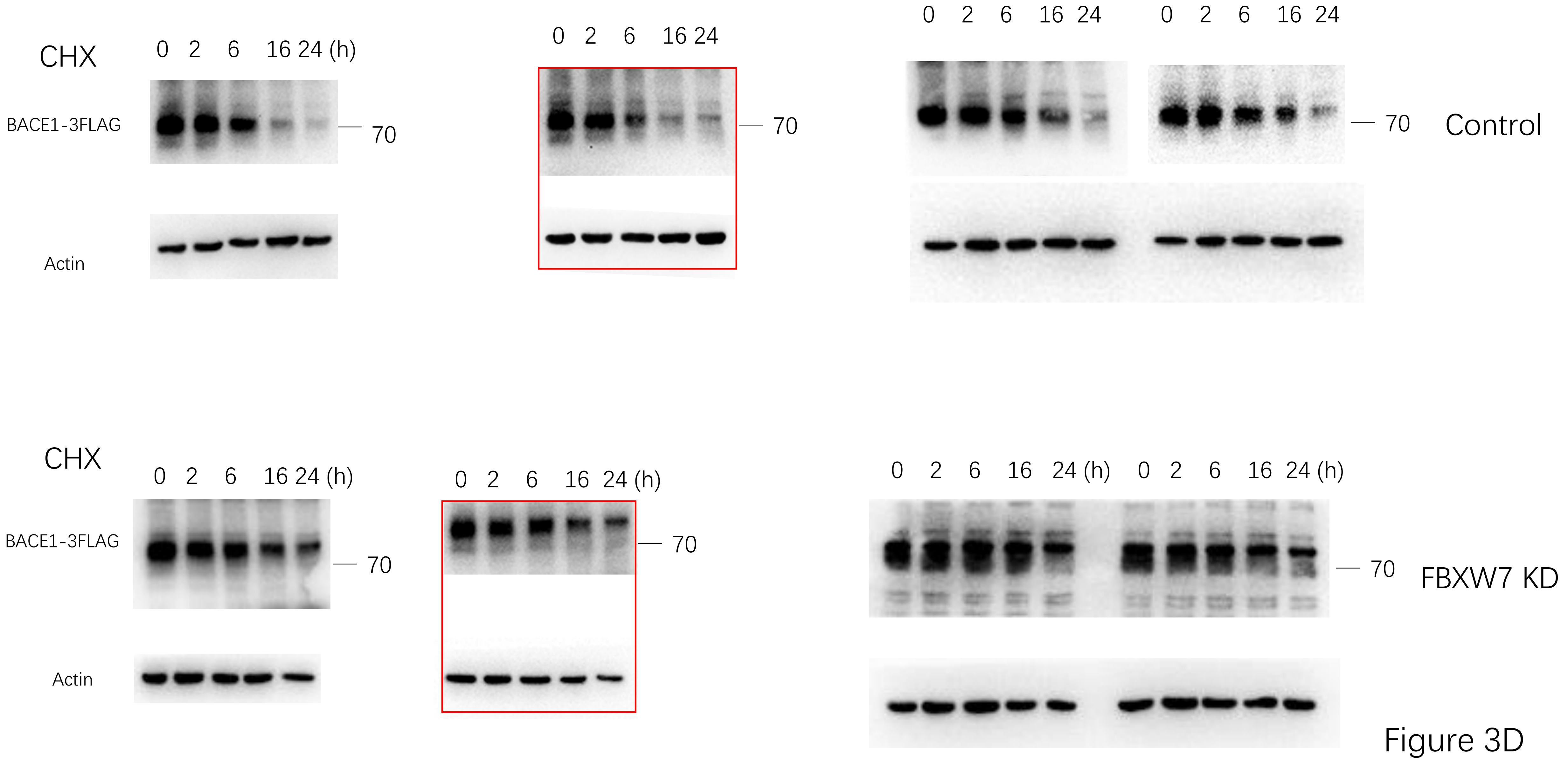

CHX
0 2 6 16 24 (h)
BACE1-3FLAG
70
Actin
0 2 6 16 24
70
0 2 6 16 24
0 2 6 16 24
70
Control
CHX
0 2 6 16 24 (h)
BACE1-3FLAG
70
Actin
0 2 6 16 24 (h)
70
0 2 6 16 24 (h)
0 2 6 16 24 (h)
70
FBXW7 KD

Figure 3D

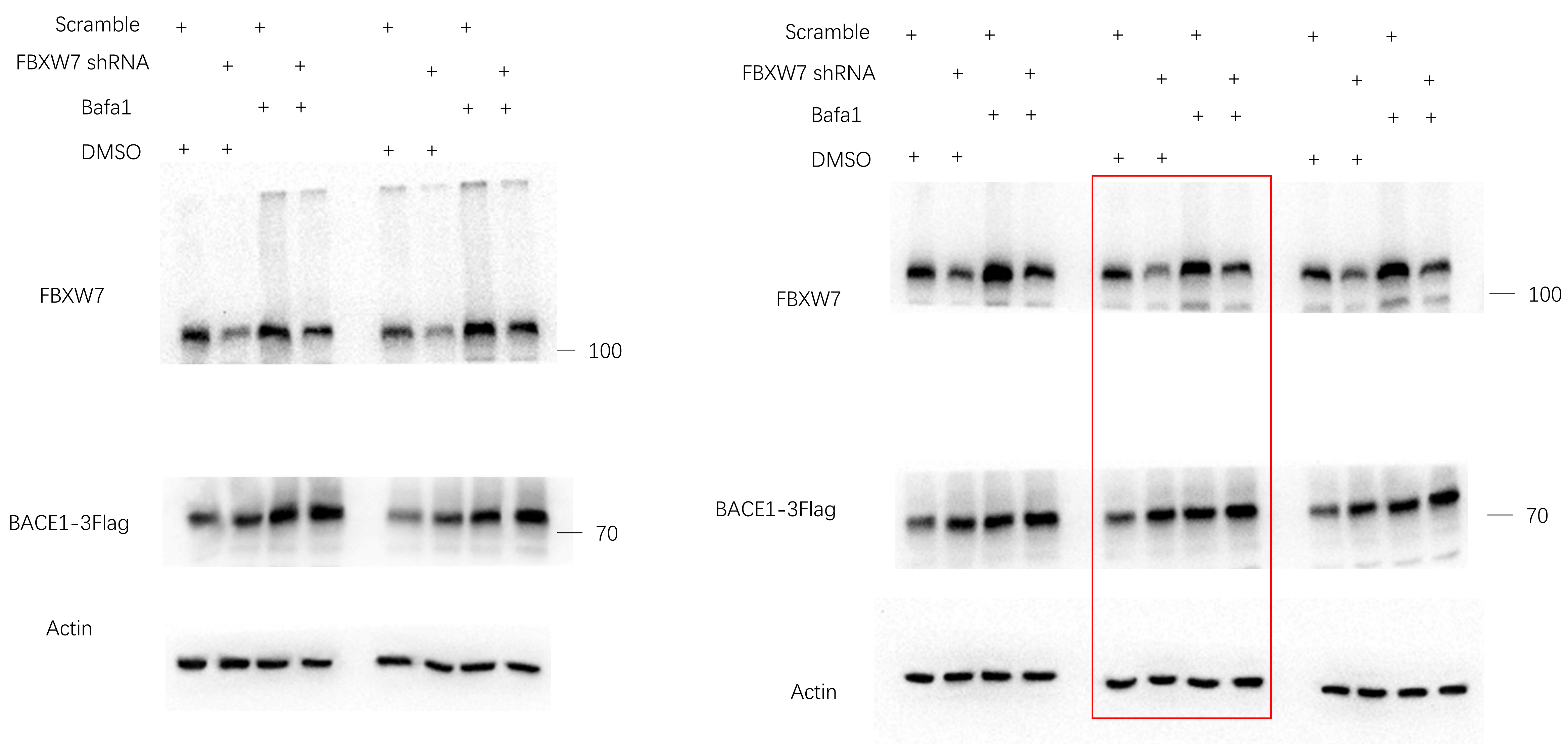

Scramble
FBXW7 shRNA
Bafa1
DMSO
FBXW7
100
BACE1-3Flag
70
Actin
Scramble
FBXW7 shRNA
Bafa1
DMSO
FBXW7
100
BACE1-3Flag
70
Actin

Figure 3F

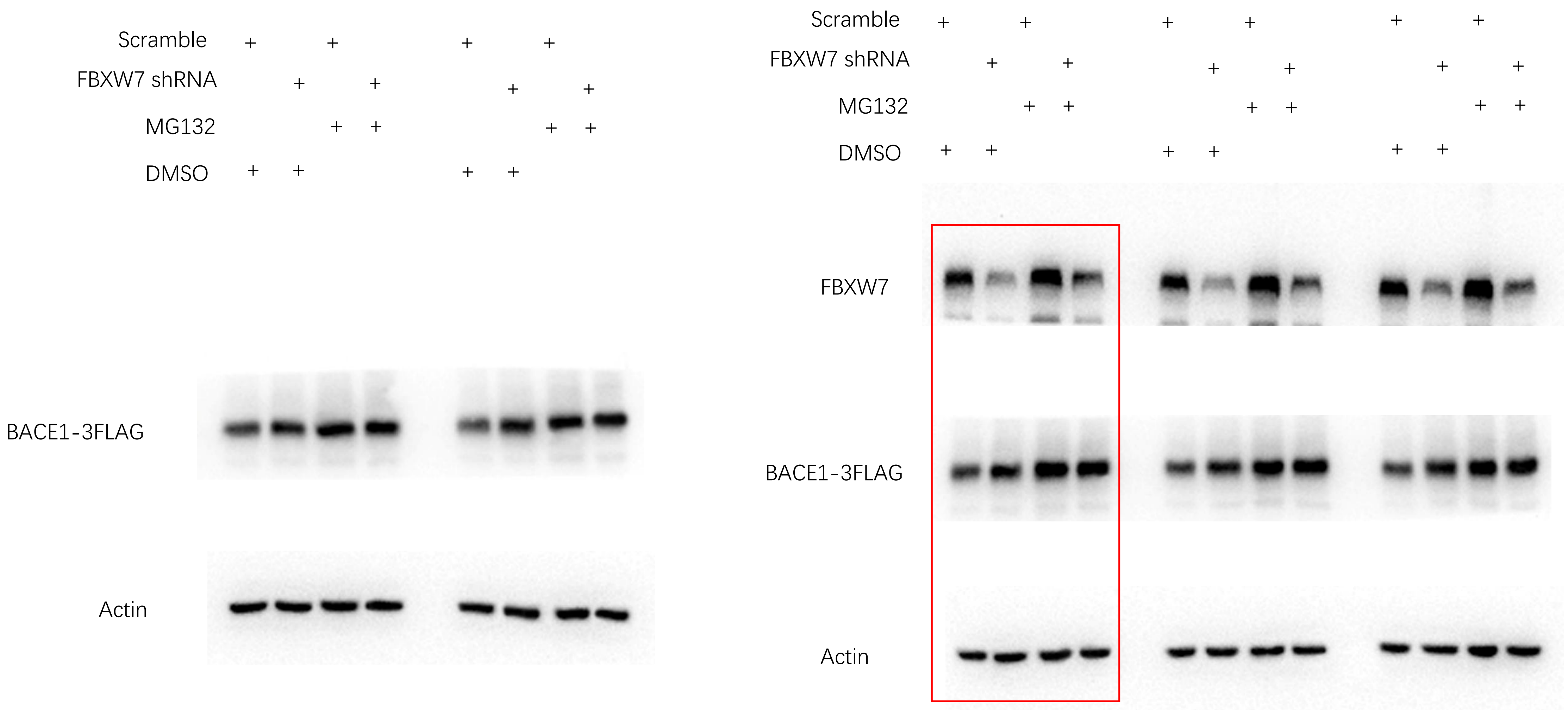
Scramble
FBXW7 shRNA
MG132
DMSO
BACE1-3FLAG
Actin
Scramble
FBXW7 shRNA
MG132
DMSO
FBXW7
BACE1-3FLAG
Actin

Figure 3H

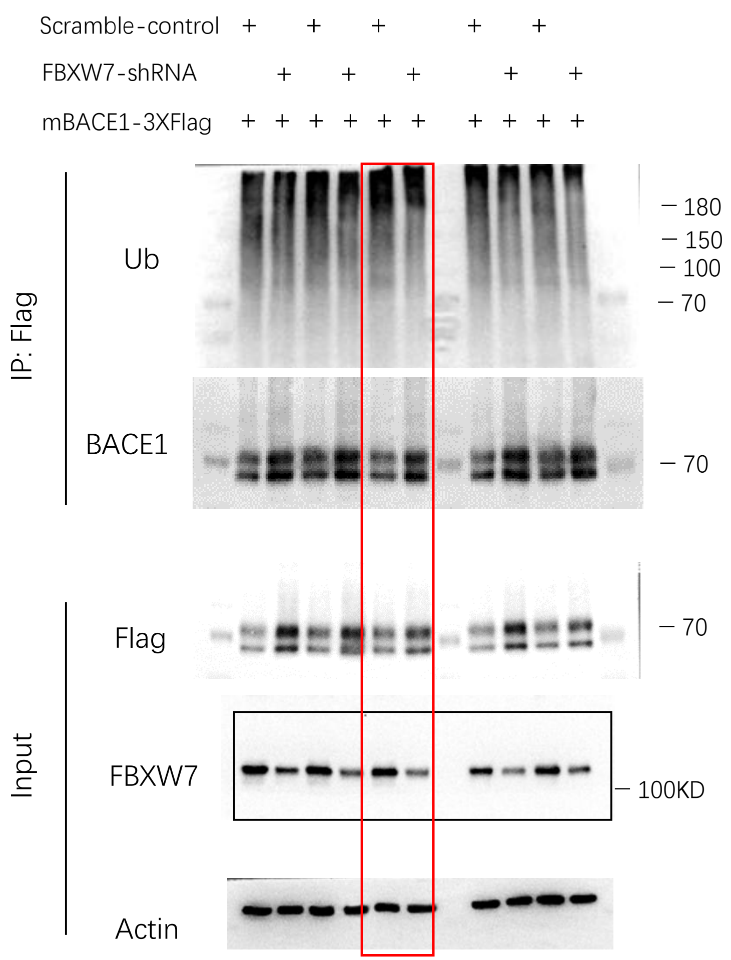
Scramble-control + + + + +
FBXW7-shRNA + + + + +
mBACE1-3XFlag + + + + + + + + + +
IP: Flag
Ub
BACE1
Input
Flag
FBXW7
Actin
180
150
100
70
70
70
100KD

Figure 3J

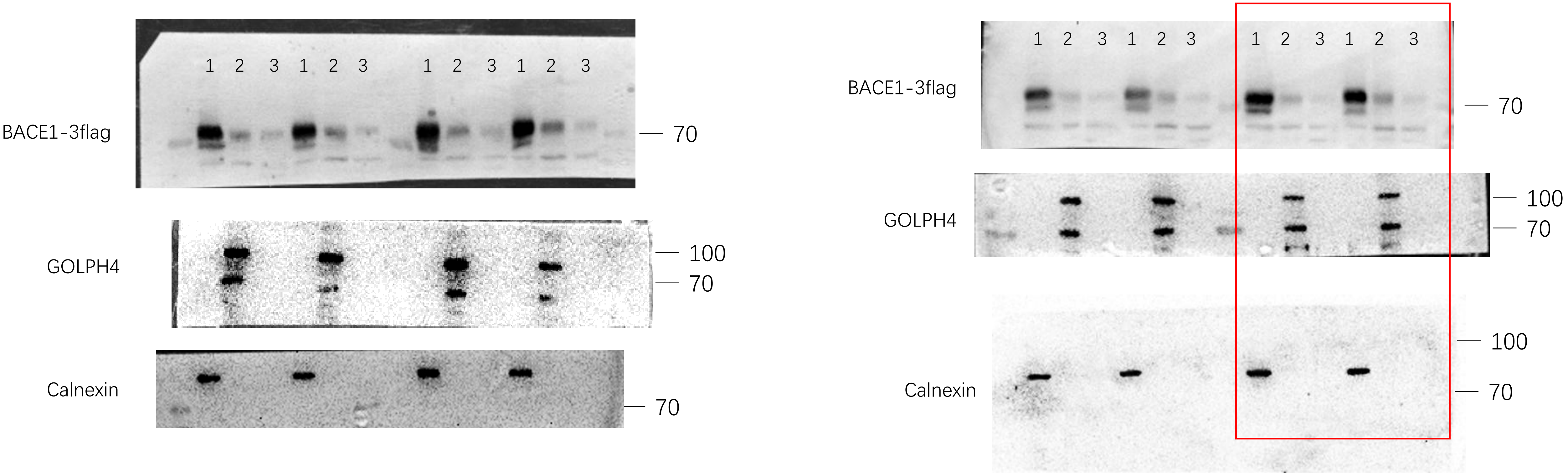

BACE1-3flag
1 2 3 1 2 3 1 2 3 1 2 3
70
GOLPH4
100
70
Calnexin
70
BACE1-3flag
1 2 3 1 2 3 1 2 3 1 2 3
70
GOLPH4
100
70
Calnexin
100
70

Figure 4A

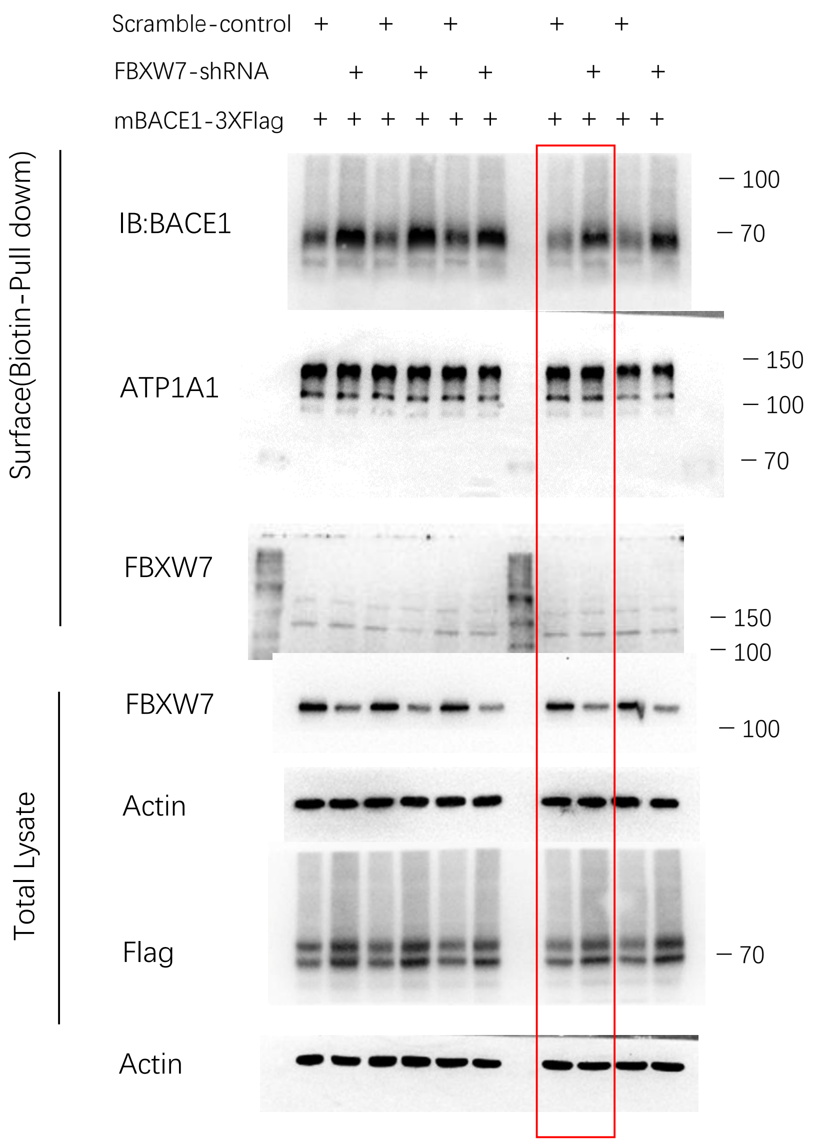
Scramble-control
FBXW7-shRNA
mBACE1-3XFlag
Surface(Biotin-Pull dowm)
IB:BACE1
ATP1A1
FBXW7
Total Lysate
FBXW7
Actin
Flag
Actin
100
70
150
100
70
150
100
100
70

Figure 4E

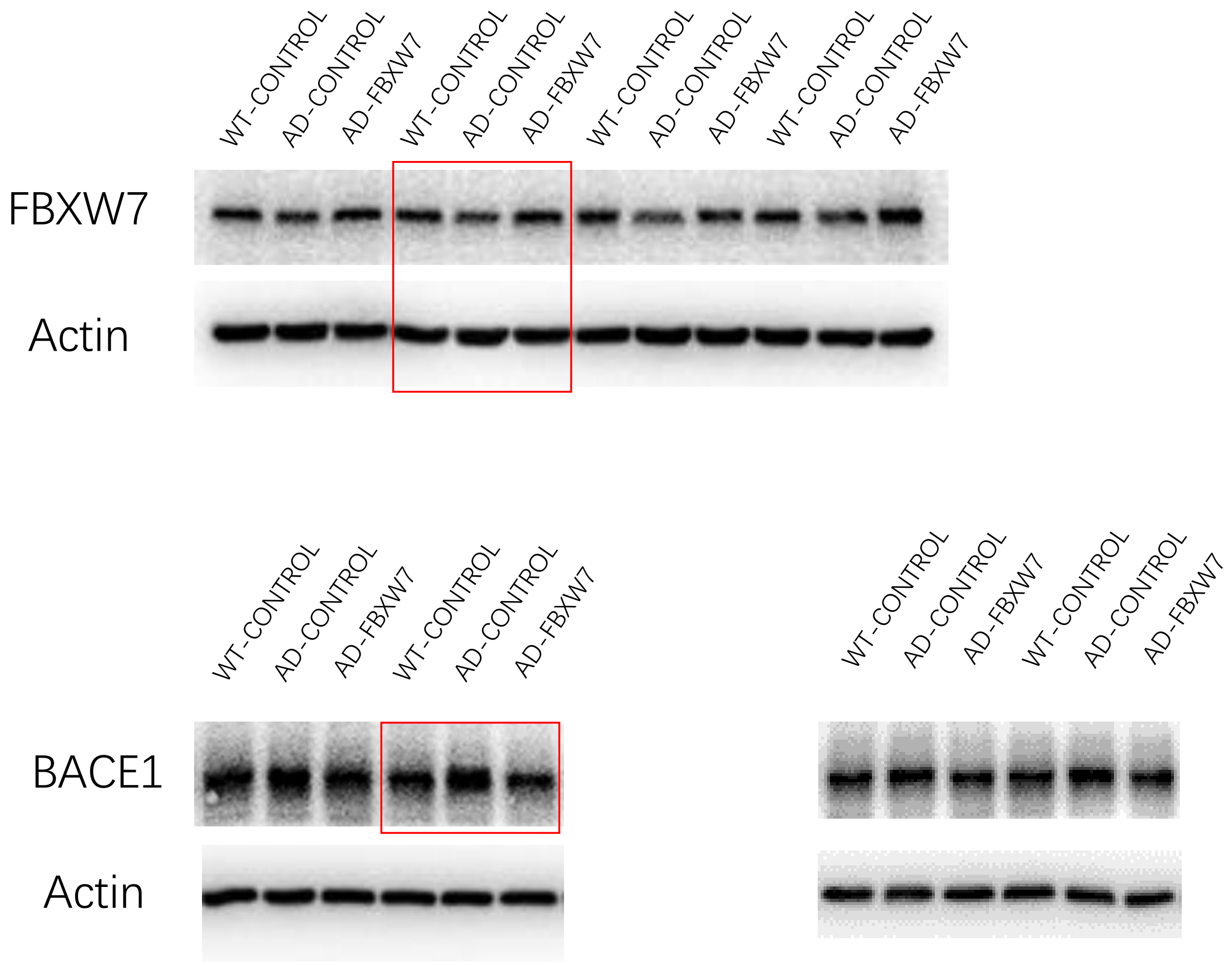

Figure 5B

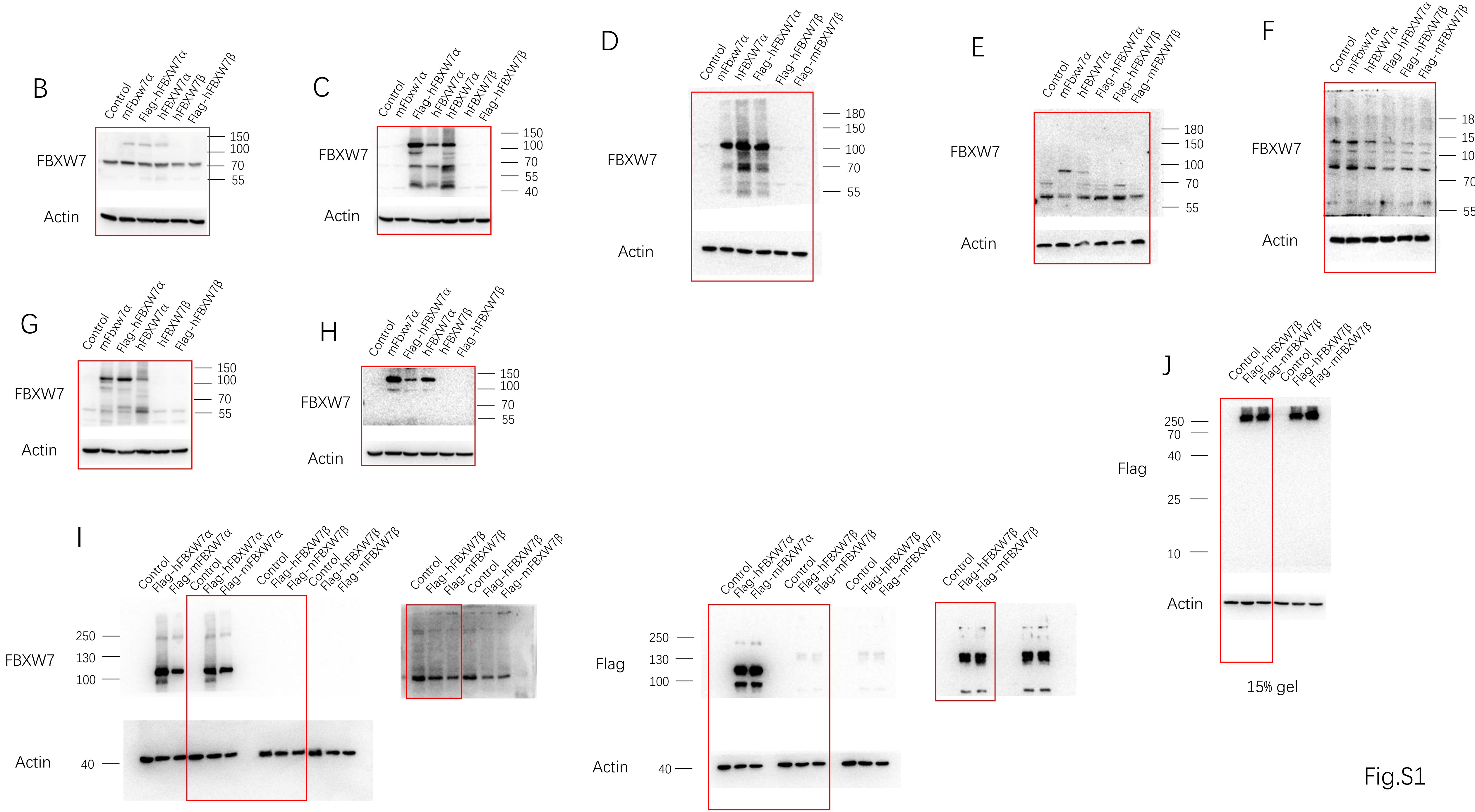
B
Control
mFbxw7α
Flag-hFBXW7α
hFBXW7α
hFBXW7β
Flag-hFBXW7β
FBXW7
Actin
150
100
70
55
C
D
E
F
G
H
I
J
180
40
250
130
25
10
Flag
Flag-mFBXW7α
Flag-mFBXW7β
15% gel

Fig.S1

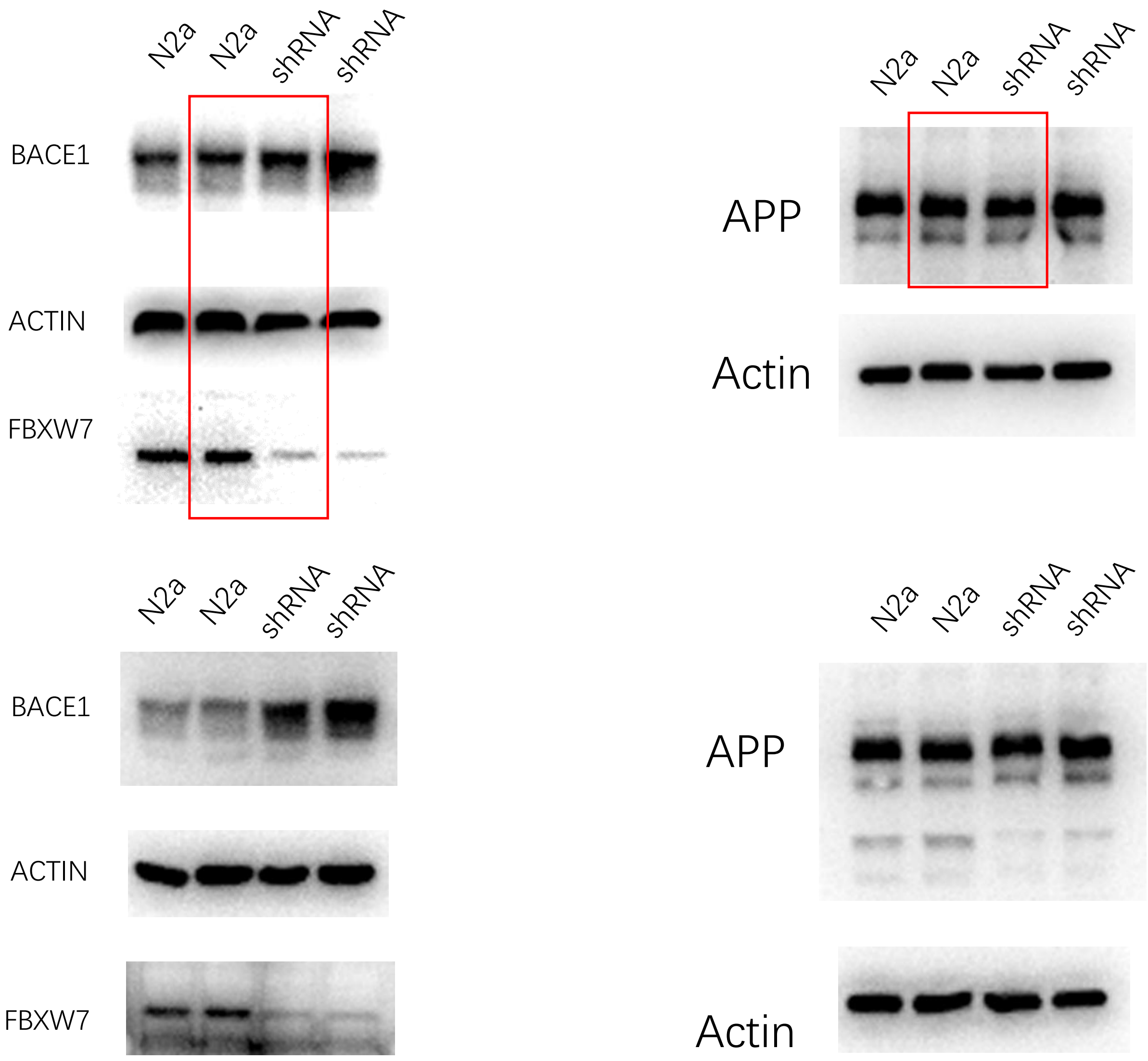

Fig.S4E

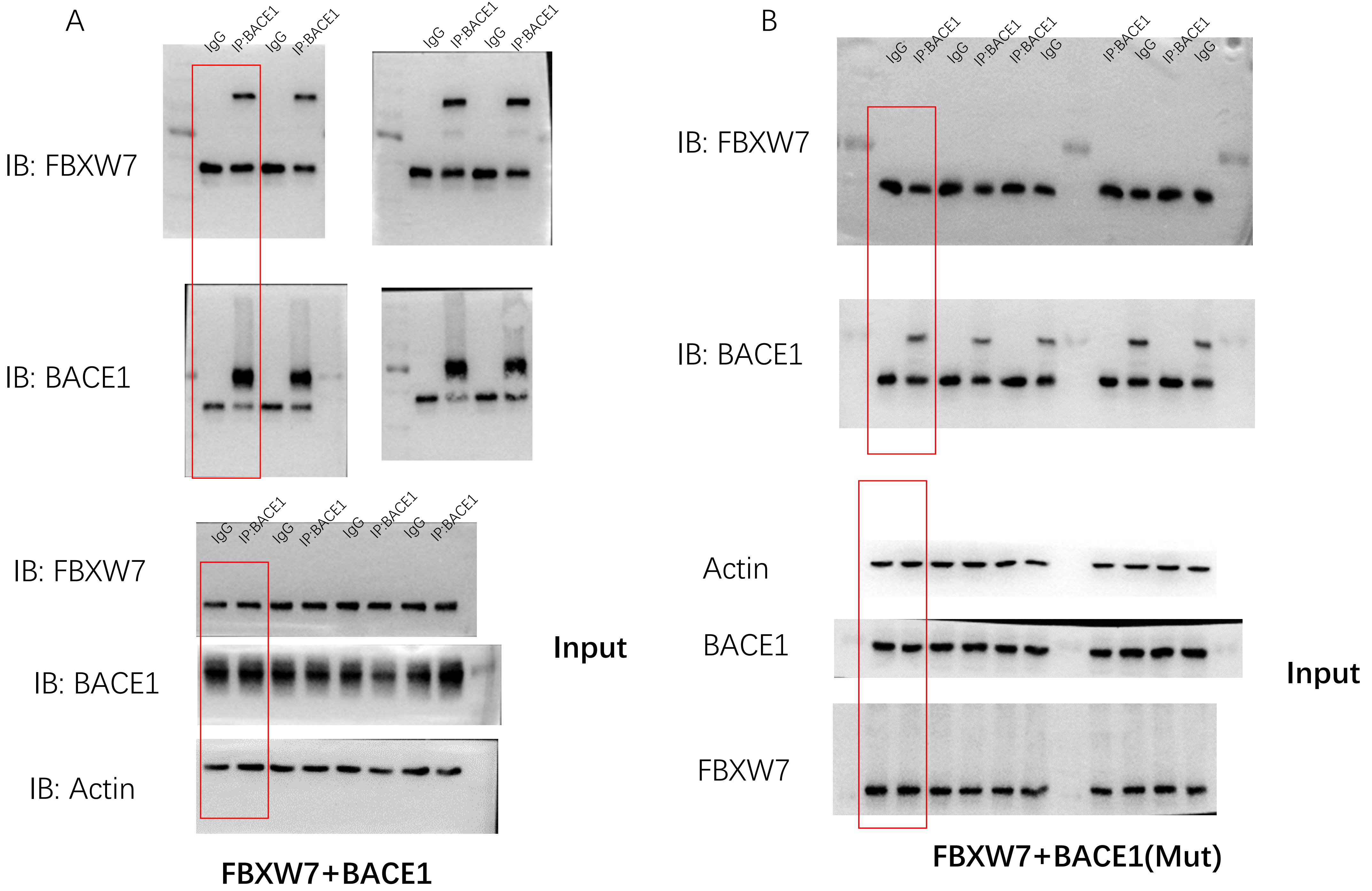
A
IgG
IP:BACE1
IgG
IP:BACE1
IgG
IP:BACE1
IgG
IP:BACE1
IB: FBXW7
IB: BACE1
IgG
IP:BACE1
IgG
IP:BACE1
IgG
IP:BACE1
IgG
IP:BACE1
IB: FBXW7
IB: BACE1
IB: Actin
Input
FBXW7+BACE1
B
IgG
IP:BACE1
IgG
IP:BACE1
IP:BACE1
IgG
IP:BACE1
IgG
IP:BACE1
IgG
IB: FBXW7
IB: BACE1
Actin
BACE1
FBXW7
Input
FBXW7+BACE1(Mut)

Fig.S5

C

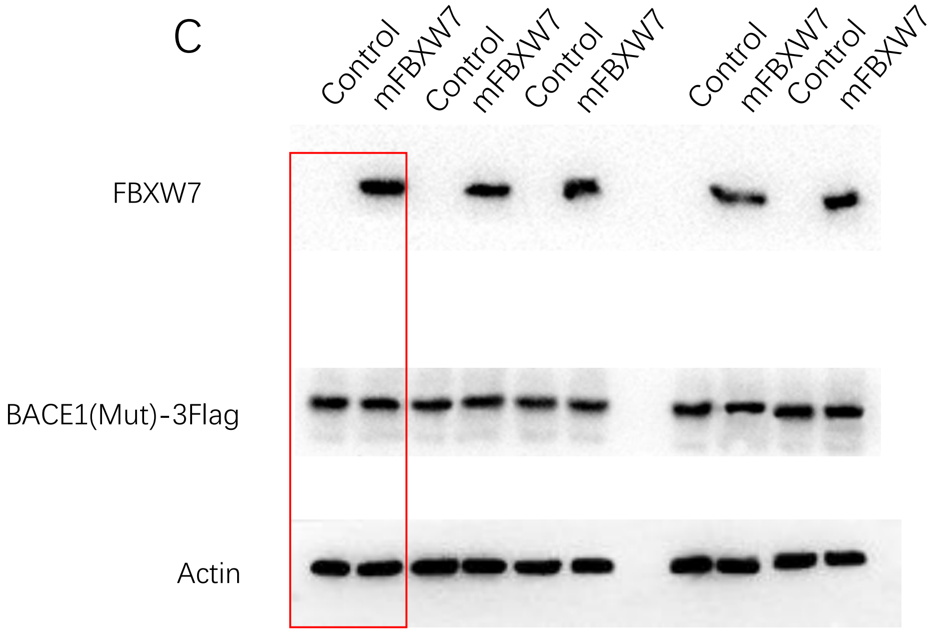
Control
mFBXW7
Control
mFBXW7
Control
mFBXW7
Control
mFBXW7
Control
mFBXW7
FBXW7
BACE1(Mut)-3Flag
Actin

E

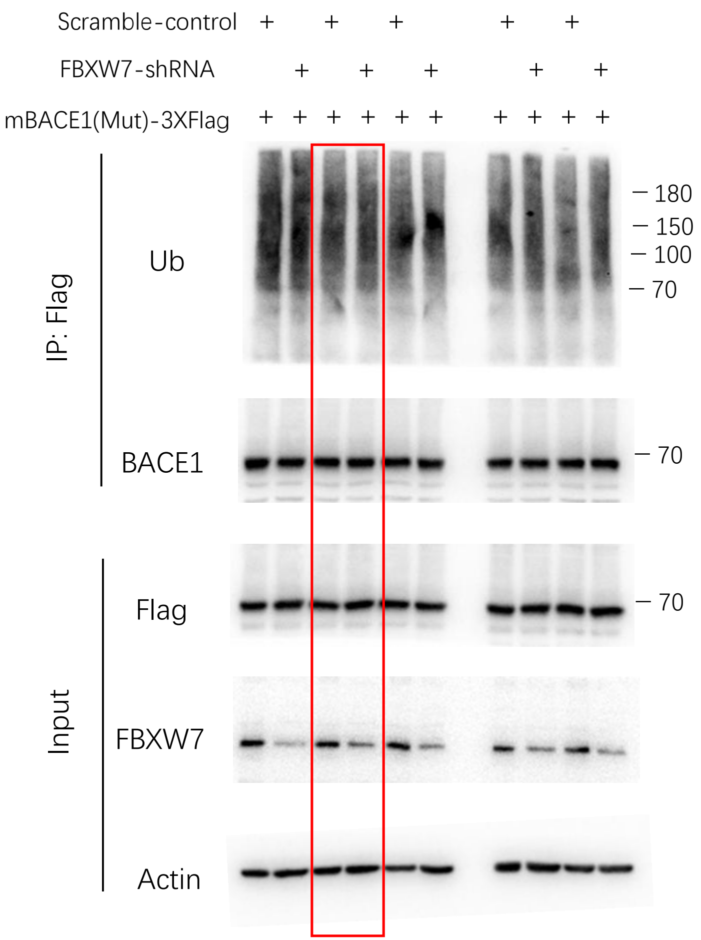
Scramble-control
FBXW7-shRNA
mBACE1(Mut)-3XFlag
IP: Flag
Ub
BACE1
Input
Flag
FBXW7
Actin
180
150
100
70
70
70

Fig.S5

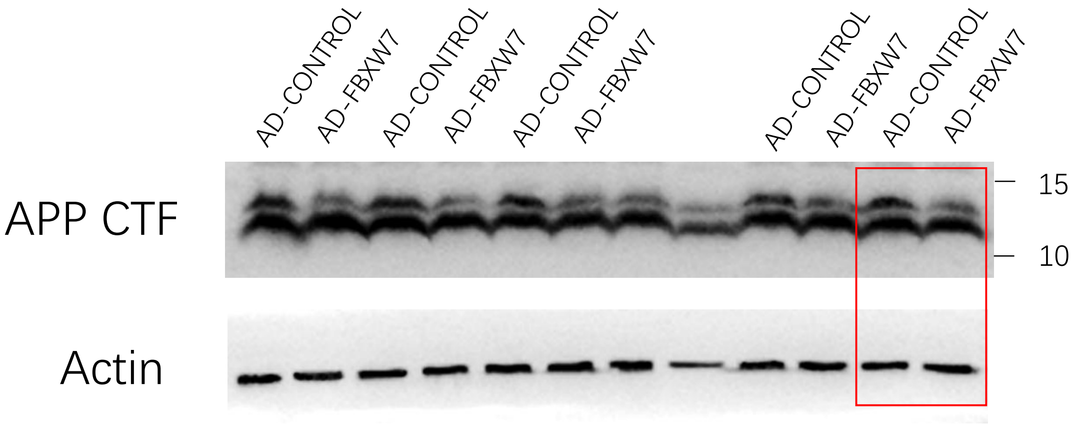
AD-CONTROL
AD-FBXW7
AD-CONTROL
AD-FBXW7
AD-CONTROL
AD-FBXW7
AD-CONTROL
AD-FBXW7
AD-CONTROL
AD-FBXW7
APP CTF
Actin
15
10

Fig.S5
