# Supplementary material for: FBXW7α regulates amyloid pathology by mediating ubiquitination and degradation of BACE1 in Alzheimer’s disease
Source: Cell Death Discov. 2026 May 20;12:300. doi: 10.1038/s41420-026-03159-y (PMC13358112; doi:10.1038/s41420-026-03159-y)
Supplement: Supplementary file 2 — Supplement Figures and captions [file 41420_2026_3159_MOESM2_ESM.pdf]

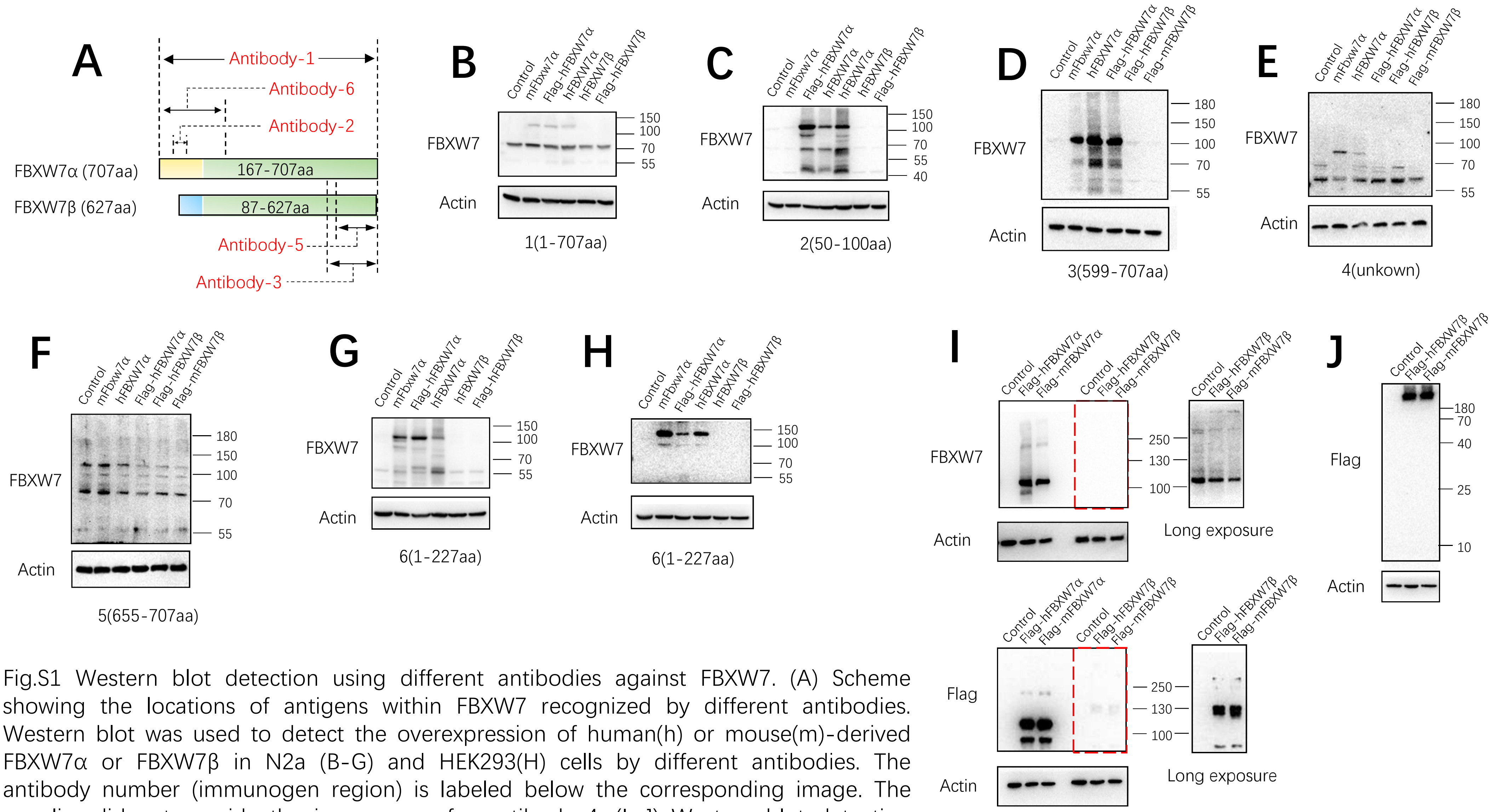

Fig.S1 Western blot detection using different antibodies against FBXW7. (A) Scheme showing the locations of antigens within FBXW7 recognized by different antibodies. Western blot was used to detect the overexpression of human(h) or mouse(m)-derived FBXW7α or FBXW7β in N2a (B-G) and HEK293(H) cells by different antibodies. The antibody number (immunogen region) is labeled below the corresponding image. The supplier did not provide the immunogen for antibody 4. (I, J) Western blot detection against either the Flag tag or FBXW7 in N2a cells overexpressing Flag tagged FBXW7α FBXW7β.

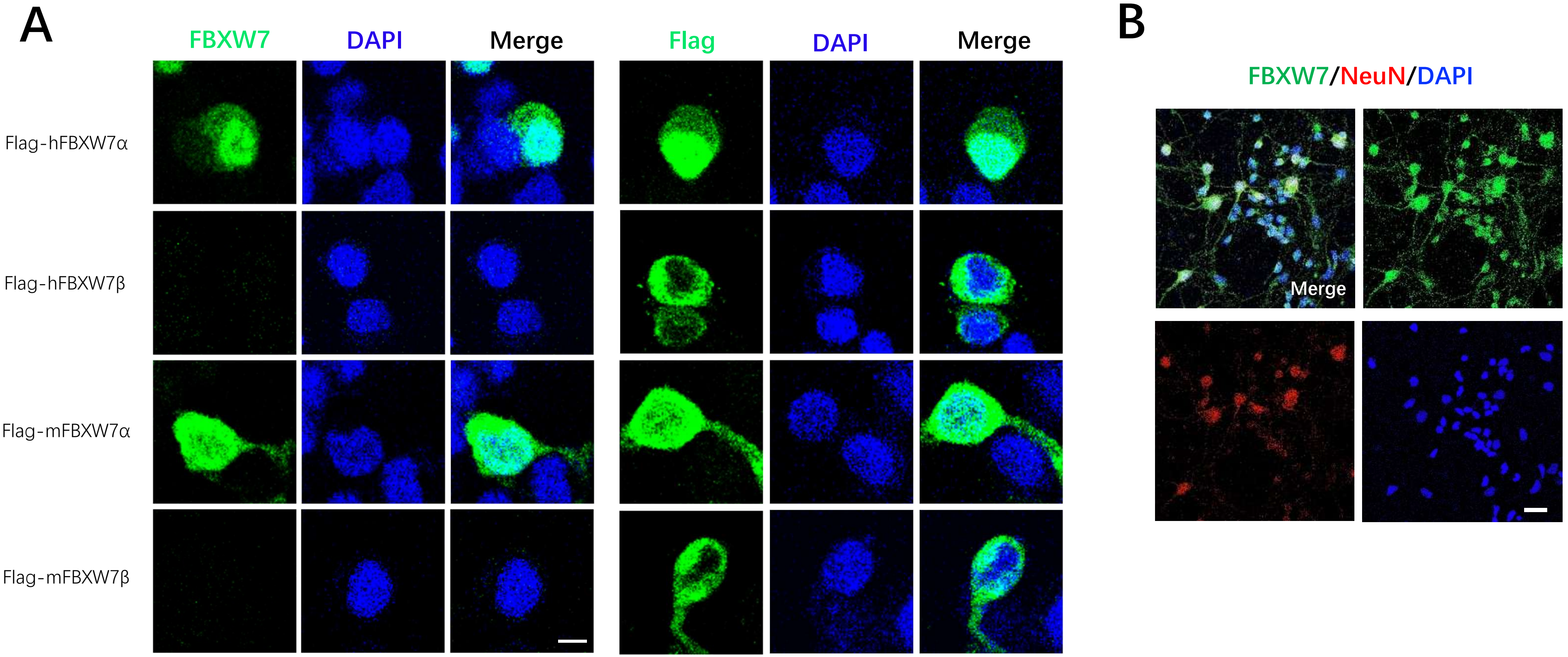

Fig.S2 FBXW7α locates in both the nucleus and cytoplasm. (A) Immunofluorescence detection of FBXW7 location using antibodies against either the Flag tag or FBXW7 in N2a cells overexpressing corresponding genes. Scale bar: 10 μm. (B) Immunofluorescence analysis of FBXW7α expression in primary neurons of wild-type mice. Scale bar: 20 μm

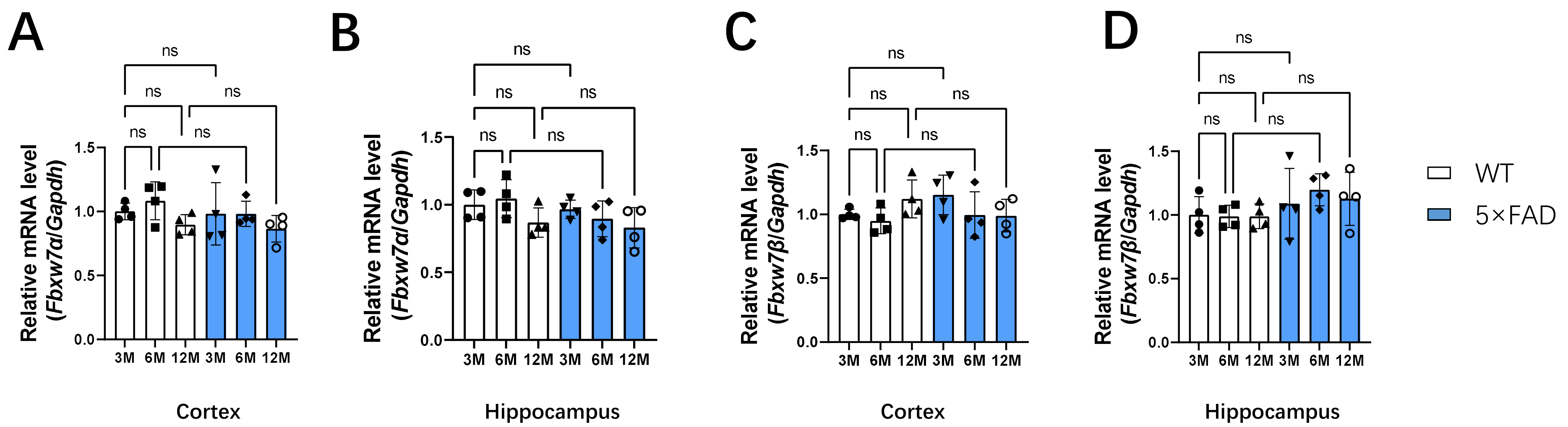

Fig.S3 RT-PCR analysis detecting mRNA level of *FBXW7α* (A-B) and *FBXW7β* (C-D) in cortical and hippocampal tissues of WT and 5×FAD mice at 3, 6, and 12 months of age (n=4 per group). Data show mean ± S.D, and each experiment was conducted with a minimum of three replicates. P value was calculated by one-way ANOVA with Bonferroni correction. ns, not significant.

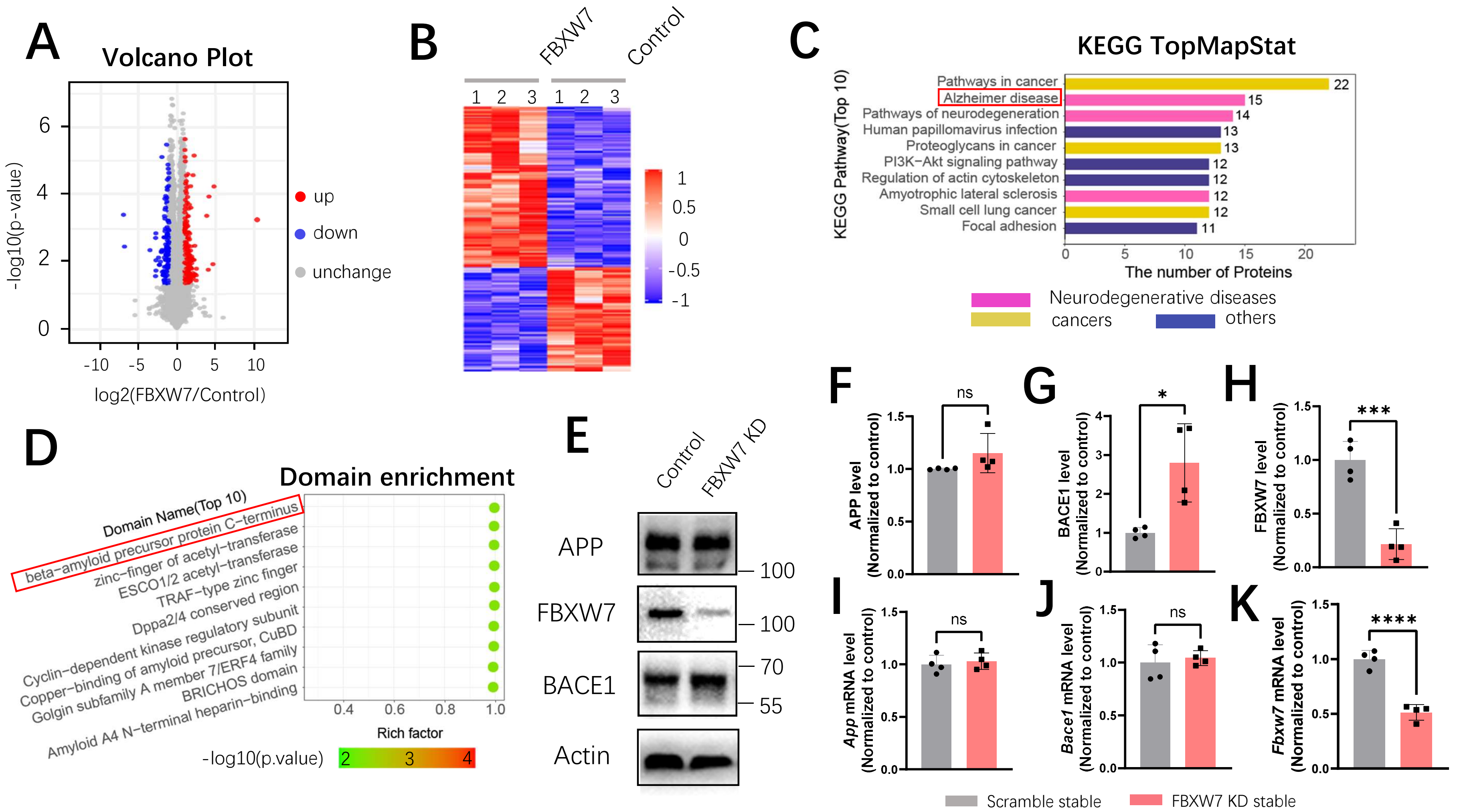

Fig.S4 Control and FBXW7α overexpressing Neuro-2a (N2a) cells (n=3) was subjected to whole proteomic analysis. (A) Valcano plot showing proteins changed (two-fold downregulated or upregulated, P value<0.05). (B) The heatmap visualizes the expression patterns of the proteins exhibiting significant expression changes in FBXW7α overexpressing cells. Red denotes upregulation while blue indicates downregulation. KEGG database (C) and protein domain (D) analysis of the differentially expressed proteins. Representative immunoblotting images (E) and quantitative analysis of APP(F), BACE1(G), and FBXW7α(H) in scramble control and FBXW7-knockdown stable N2a cell line (n=4 per group). RT-PCR analysis of *App*(I), *Bace1*(J), and *Fbxw7α*(K) genes in scramble control and FBXW7-knockdown stable N2a cell line (n=4 per group). Data show mean ± S.D. P values were determined by Student's t test. *P < 0.05; ***P < 0.001; ****P < 0.0001; ns, not significant.

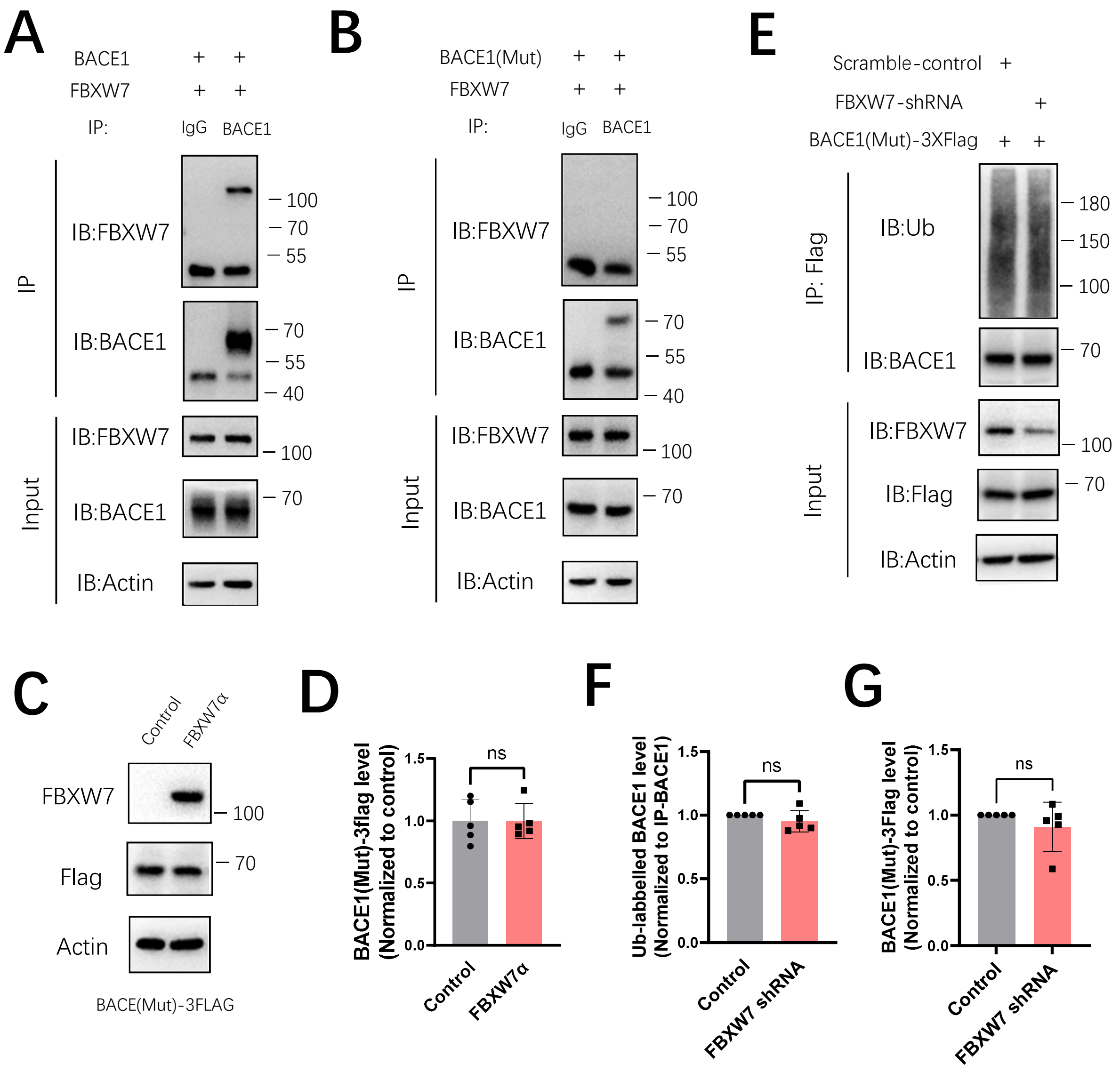

Fig.S5 The role of the FBXW7 recognition motif in the ubiquitination of BACE1. Co-immunoprecipitation assays were performed to detect the interaction of FBXW7 with BACE1(A) or mutant BACE1 (B). Representative immunoblotting images (C) and quantitative analysis (D) of mutant BACE1-3Flag level upon FBXW7α overexpression. (E-G) Immunoprecipitation detection of ubiquitinated (E,F) and total (E,G) mutant BACE1-3Flag protein levels in N2a cells upon FBXW7 knockdown. Data show mean ± S.D, and each experiment was conducted with a minimum of three replicates. (n=5 per group). P value was calculated by Student's t test.ns, not significant.

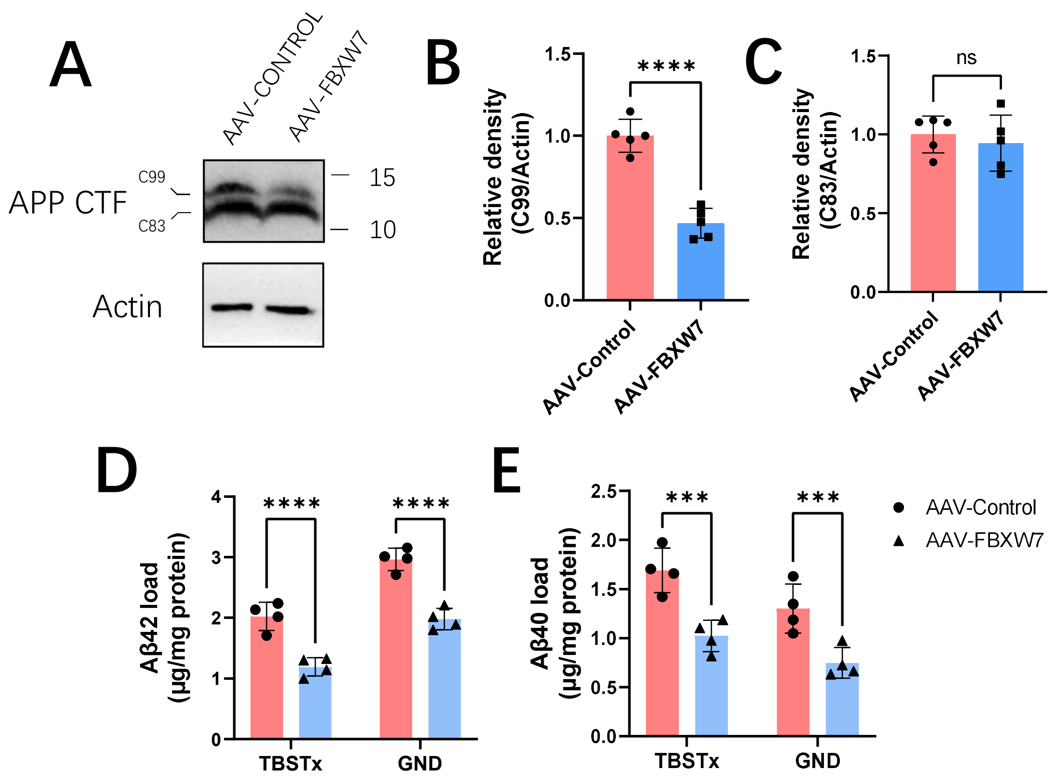

Fig.S6 Restoration of FBXW7α expression reduces Aβ production in the hippocampus of 5×FAD mice. Representative immunoblotting images (A) and quantitative analysis of C99(B), and C83(C) in 5×FAD mice injected by FBXW7α expressing or vector control AAVs in the hippocampi (n=5 per group). Quantification of detergent soluble (TBSTx) and insoluble (GND) Aβ42 (D) and Aβ40 (E) in the hippocampi injected by corresponding AAVs (n=4 per group).. Data show mean ± S.D, and each experiment was conducted with a minimum of three replicates. P value was calculated by Student's t test. ***p value < 0.001, ****p value < 0.0001, not significant.
