# Supplementary material for: FBXW7α regulates amyloid pathology by mediating ubiquitination and degradation of BACE1 in Alzheimer’s disease
Source: Cell Death Discov. 2026 May 20;12:300. doi: 10.1038/s41420-026-03159-y (PMC13358112; doi:10.1038/s41420-026-03159-y)
Supplement: Supplementary file 3 — Supplement table [file 41420_2026_3159_MOESM3_ESM.docx]

Table S1 qRT-PCR primers used in this study

| *App* | Forward | GACCATCCAGAACTGGTGCAAGC |
| --- | --- | --- |
|  | Reverse | GCGACGGTGTGCCAGTGAAGAT |
| *Bace1* | Forward | GCGTGCCAACATTGCTGCCATCAC |
|  | Reverse | CTGGTTGAGGGGGAAGCCAGC |
| *Fbxw7α* | Forward | GACCTGCCCGCTCACCAGC |
|  | Reverse | GTTGCTGAACATGGTACAAGGCCAG |
| *Fbxw7β* | Forward | GGCACAGAATCCCTGAAGGGG |
|  | Reverse | GTTGCTGAACATGGTACAAGGCCAG |

All the sequences are showed as 5’ to 3’.
